# Knockdown of hepatic mitochondrial calcium uniporter mitigates MASH and fibrosis in mice

**DOI:** 10.1186/s13578-024-01315-4

**Published:** 2024-11-10

**Authors:** Shuyu Li, Fangyuan Chen, Min Liu, Yajun Zhang, Jingjing Xu, Xi Li, Zhiyin Shang, Shaoping Huang, Shu Song, Chuantao Tu

**Affiliations:** 1 Department of Gastroenterology and Hepatology, Zhongshan Hospital, Fudan University, Shanghai, 200032 China; 2 Department of Gastroenterology, Shanghai Public Health Clinical Center, Fudan University, Shanghai, 201508 China; 3 Department of Pathology, Shanghai Public Health Clinical Center, Fudan University, Shanghai, 201508 China; 4 Department of Geriatrics, Zhongshan Hospital, Fudan University, Shanghai, 200032 China

**Keywords:** MCU, Steatohepatitis, Liver fibrosis, Mitochondrial dysfunction, Oxidative stress

## Abstract

**Background:**

Mitochondrial calcium uniporter (MCU) plays pleiotropic roles in cellular physiology and pathology that contributes to a variety of diseases, but the role and potential mechanism of MCU in the pathogenesis of metabolic dysfunction-associated steatohepatitis (MASH) remain poorly understood.

**Methods and results:**

Here, hepatic knockdown of MCU in C57BL/6J mice was achieved by tail vein injection of AAV8-mediated the CRISPR/Cas9. Mice were fed a Choline-deficient, L-amino acid-defined high-fat diet (CDAHFD) for 8 weeks to induce MASH and fibrosis. We find that expression of MCU enhanced in MASH livers of humans and mice. MCU knockdown robustly limits lipid droplet accumulation, steatosis, inflammation, and hepatocyte apoptotic death during MASH development both in vivo in mice and in vitro in cellular models. MCU-deficient mice strikingly mitigate MASH-related fibrosis. Moreover, the protective effects of MCU knockdown against MASH progression are accompanied by a reduced level of mitochondrial calcium, limiting hepatic oxidative stress, and attenuating mitochondrial dysfunction. Mechanically, RNA sequencing analysis and protein immunoblotting indicate that knockdown MCU inhibited the Hippo/YAP pathway activation and restored the AMP-activated protein kinase (AMPK) activity during MASH development both in vitro and in vivo.

**Conclusions:**

MCU is up-regulated in MASH livers in humans and mice; and hepatic MCU knockdown protects against diet-induced MASH and fibrosis in mice. Thus, targeting MCU may represent a novel therapeutic strategy for MASH and fibrosis.

**Graphical Abstract:**

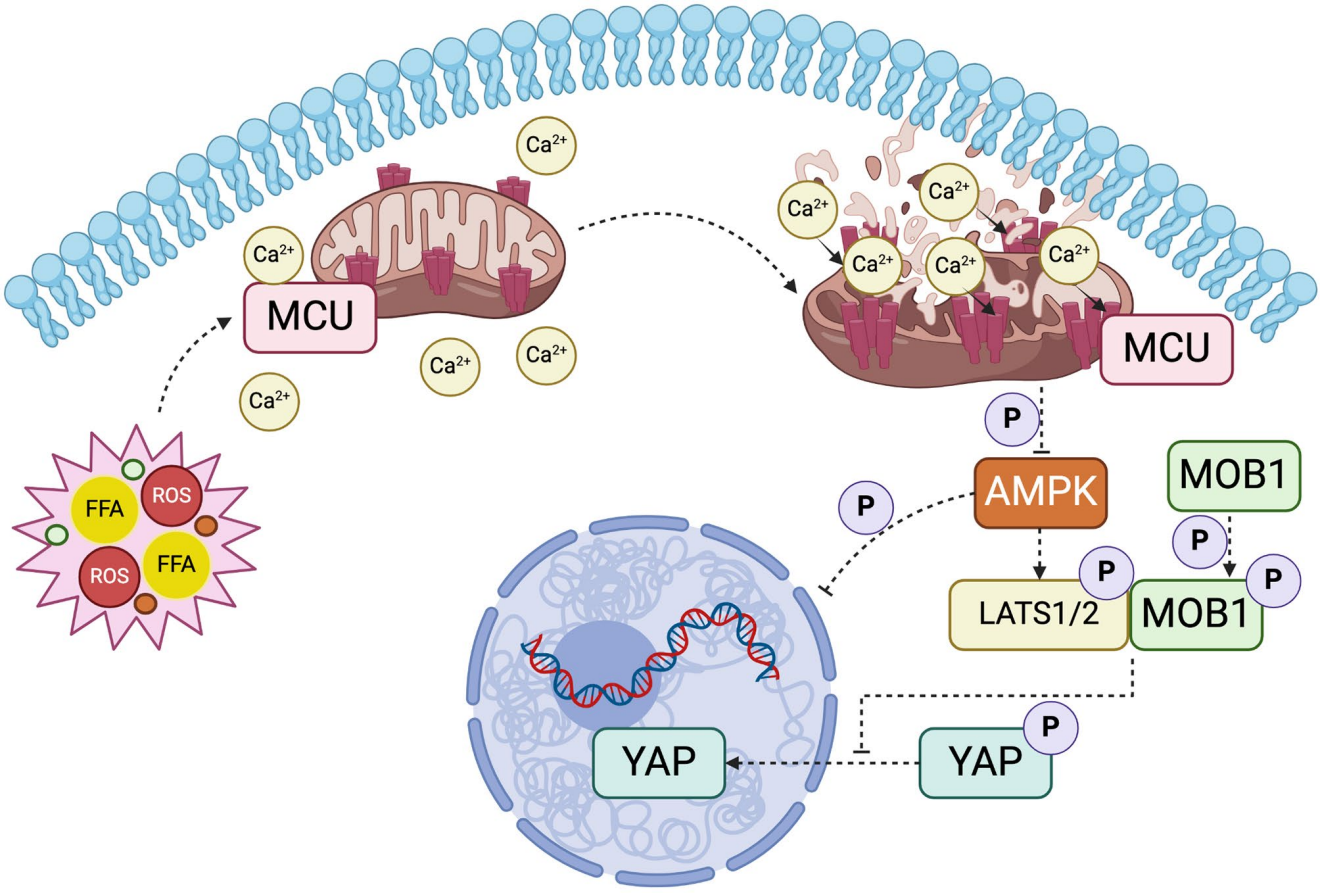

**Supplementary Information:**

The online version contains supplementary material available at 10.1186/s13578-024-01315-4.

## Introduction

Metabolic dysfunction-associated steatohepatitis (MASH), formerly known as nonalcoholic steatohepatitis (NASH), is a severe pathologic stage of metabolic dysfunction-associated steatotic liver disease (MASLD) with hepatic necroinflammation and different degrees of fibrosis [1–3]. MASLD is a more accurate and more appropriate name instead of the previous non-alcoholic fatty liver disease (NAFLD) that reflects its association with metabolic syndrome and conditions including obesity, type 2 diabetes mellitus, and high cholesterol [1–3]. In particular, MASH fibrosis can gradually progress to cirrhosis, and trigger hepatocellular carcinoma (HCC) and liver failure. Thus, liver fibrosis is the strongest determinant of cause-specific mortality in patients with progressive MASH [4]. Despite its striking global increase and the importance of MASLD/MASH, there are still no approved agents for treating or reversing this metabolic disorder.

The pathologic mechanism for MASH is complex and multifactorial, which has been explained by the ‘multiple parallel hits’ hypothesis, including obesity, insulin resistance, lipotoxicity, oxidative stress, mitochondrial dysfunction, endoplasmic reticulum (ER) [5–7]. Recent evidence has shown that the calcium (Ca^2+^) signaling in hepatocytes is a complex and dynamic means of communication that controls a diverse array of cellular functions, such as cell survival and apoptosis, oxidative metabolism, exocytosis, mitochondrial function, and ER stress [8–12]. Moreover, it has been established that mitochondria are active participants in cellular Ca^2+^ signaling crucial for cell life and death [10–12]. Particularly, mitochondrial Ca^2+^ flux is a critical regulator of hepatic metabolism, homeostasis, gene transcription, and cell bioenergetics [11, 12]. On the contrary, impaired Ca^2+^ signaling in hepatocytes has notably been associated with obesity, T2DM, and MASLD [8–10]. Thus, targeting hepatic Ca^2+^ signaling might serve as a therapeutic strategy for metabolic liver diseases including MASFD and MASH.

Recently, it has been demonstrated that mitochondrial Ca^2+^ uniporter (MCU), located in the inner mitochondrial membrane (IMM), is a highly selective ion channel that functions as mitochondrial Ca^2+^ uptake [13–16]. MCU channel functions as a heteromeric protein complex of ~ 450–800 kDa that includes the ion-conducting core MCU protein and several MCU-associated regulatory proteins, including MCU dominant negative beta subunit (MCUb), essential MCU regulator (EMRE), the mitochondrial Ca^2+^ uptake (MICU) family, MCU regulator 1 (MCUR1), and solute carrier 25A23 [15–18]. Furthermore, MCU plays a critical determinant role in cell fate and governs respiration, mitophagy/autophagy, and the mitochondrial pathway of apoptotic cell death [10–14].

An emerging body of evidence demonstrates that MCU plays a pleiotropic role in physiology and pathology that mediates a variety of diseases such as neurological disorders, cancer, cardiomyopathies, pulmonary fibrosis, muscle hypertrophy, and liver pathogenesis [17–25]. Noteworthy, several recent studies have demonstrated that MCU is also involved in lipid metabolism [8–10]; and lipid accumulation in hepatocytes substantially altered intracellular Ca^2+^ homeostasis that further enhanced the hepatosteatosis and thereby triggered MASLD progression to HCC [25–28]. However, the precise role and mechanism of the MCU in the pathogenesis of MASH and fibrosis remains largely unknown. We hypothesize that MCU-mediated Ca^2+^ signaling plays a pathological role in MASH and fibrosis and may represent a promising therapeutic target for this disorder.

## Methods

### Reagents and antibodies

Reagents in this study were obtained as follows. Antimycin A (#1397-94-0), BODIPY 493/503 (#D2191), Mitochondrial Superoxide Indicators (MitoSOX, #M36008), and Hoechst (#62249) from Thermo Fisher Scientific (Boston, USA); Oliec acid (#O1383), Palmitic acid (#P9767), and RIPA buffer (#R0278) from Sigma-Aldrich (Missouri, USA); Albumin BovineV (fatty acid-free; #A8850) from Solarbio (Beijing, China); Dihydroethidium (DHE; #S0063), ROS Assay Kit (#S0033S), Lipid Peroxidation MDA Assay Kit (#S0131), Catalase Assay Kit (#S0051), Puromycin (#ST551), and DAPI (#C1005) from Beyotime (Shanghai, China); Oligomycin (#S1479), ADP (#S9368), Succinic acid (#S3791), Glutamic acid (#S6266), Malic acid (#S9001), FCCP (#S8276), Rotenone (#S2348), and Dorsomorphin (#BML-275) from Selleck Chemicals (Houston, TX, USA); Rhod-2 AM (#HY-D0989) from MedChemExpress (New Jersey, USA). Penicillin, Streptomycin, and trypsin-ethylenediaminetetraacetic acid from Genom Biotechnology (Shanghai, China); and ATP Luminescent kit (#40210ES10) from Yeasen Biotechnology (Shanghai, China). All antibodies in this study are listed in Table S1.

### Animals, diets, and experimental design

Six-week-old Male C57BL/6J wild-type (WT) mice (Shanghai Slack laboratory Animal Co., Ltd., Shanghai, China) lived in an SPF condition. Mice had unrestricted access to food and liquids with a 12-hour light-dark cycle. Mice were randomly divided into 4 groups (*n* = 9–15 mice/group). Mice were given either a normal chow diet (NCD) or a choline-deficient, L-amino acid-defined, high-fat diet (CDAHFD; Research Diets, USA) for 8 weeks [29, 30]. Conditional MCU knockdown mice were using adeno-associated virus serotype 8 (AAV8). For the CRISPR/Cas9 studies, All-in-One AAV8 encoding small-guide RNA (sgRNA) targeting MCU or scrambled sgRNA were provided by Hanyin Biotechnology Limited Company (Shanghai, China) (Additional file 1: Fig. S1). C57BL/6J mice were injected with either a mixture of AAV8-sgMCU or AAV8-sgCtrl at the beginning of CDAHFD-feeding. After 8 weeks, except for 3 mice in each group for use to isolate primary hepatocytes, all mice were sacrificed. Liver samples were stored at -80 °C or fixed in freshly prepared 4% paraformaldehyde.

To further verify the expression of MCU in the liver, a different diet-induced MASH mouse model was induced by feeding a high-fat/calorie diet (HFCD) for 16 weeks along with high carbohydrates (HC: 55% fructose/45% glucose) in drinking water [31]; and mice with NCD serves as the control (*n* = 8 mice/group). The Animal Care Committee of Zhongshan Hospital, Fudan University, accepted the guidelines for the care and use of laboratory animals, which were followed in all animal research (No. 2019 − 186).

### Cell cultures and treatments

From the specified animals, primary hepatocytes were extracted using the collagenase perfusion procedure that has been previously published [6]. In summary, newly extracted hepatocytes were cultivated in RPMI medium 1640 supplemented with 1.0 g/L glucose, 10% fetal bovine serum (FBS), 100 units/mL penicillin, and 100 mg/mL streptomycin at 37 °C in a humidified chamber. The hepatocytes were seeded into collagen I-coated petri plates. Six hours after plating, the medium was changed, and the cells received the appropriate treatment [32]. In this study, AML12 or primary hepatocytes were induced by oleic acid and palmitic acid (OPA) as the model of saturated fatty acid-induced lipotoxicity [33]. Briefly, 200 µM OA and 100 µM PA were first conjugated to bovine serum albumin (BSA) and then added to the medium to induce oxidative stress and hepatocellular lipotoxicity.

AML12 hepatocytes were transfected with different short hairpin (sh) RNAs as follows: non-targeted scrambled control shRNA (shScr) and 2 groups of shMCU (#1 Sequence 5’~3’ CACGTTTCGACCTAGAGAA and #2 Sequence 5’~3’ GGAGAAGGTACGAATTGAA). The shMCU and shScr lentiviral transduction particles were provided by Hanyin Biotechnology (Shanghai, China). The shMCU and shScr were synthesized and the inserted into a U6-MCS-CMV-Puro vector, and the empty vector was packaged as negative control. Then the stable transfectants were screened and cultured in medium containing puromycin. MCU knockdown was confirmed by qRT-PCR and western blotting respectively (Additional file 1: Fig. S2A and B). We finally selected shMCU (#1) with the highest efficiency for the construct of the shRNA.

Primary hepatocytes were transfected with siRNA for MCU to transiently knock down MCU. Non-targeted scrambled control siRNA (siScr) and 3 groups of siMCU were transfected in AML12 cells to verify the knockdown efficiency of siMCU and select the most efficient one. The siMCU and siScr were provided by Genepharma (Shanghai, China). The produced oligo sequences are displayed in Table S2. MCU knockdown efficiency was verified by qRT-PCR and western blotting, respectively (Additional file 1: Fig. S2C and D). The highest knockdown efficiency siMCU (#1) was finally selected to transfect primary hepatocytes.

The cDNA encoding mouse MCU (1053 bp) was produced by qRT-PCR and was subcloned into the EcoRI site of the pEX-1 (pGCMV/MCS/EGFP/Neo) expression vectors (GenePharma, Shanghai, China). AML12 hepatocytes were transfected with mouse MCU cDNA plasmids (5 µg) or the empty vector pEX-1 as a negative control for 48 h using Lipofectamine 3000 (Thermo Fisher Scientific) according to the manufacturer’s protocols. The effectiveness of genetic overexpression was confirmed by qRT-PCR and western blotting (Additional file 1: Fig. S2E and F), respectively. Then the transfected cells were incubated in DMEM/F12 medium with OPA or vehicle treatment for 24 h, cells were collected and analyzed for further testing.

### Whole-transcriptome amplification and RNA-sequencing analysis

Employing Trizol Reagent (Thermo Fisher), total RNA was extracted from liver tissues and utilized for RNA-seq analysis. On an Apollo Library Prep System (Illumina, San Diego, CA), library preparation was carried out using the TruSeq Stranded mRNA Sample Prep Kit (Takara, Shiga, Japan). Liver RNA-seq was performed on an Illumina Novaseq™ 6000 platform in 2 × 150 bp paired-end sequencing (LC-Bio Technology, Hangzhou, China). We used HISAT2 (https://daehwankimlab.github.io/hisat2/, version: hisat2-2.2.1) package to map the clean reads to each gene and normalized the raw data to Fragments Per Kilobase of exon model per Million mapped fragments for subsequent analyses. Bioinformatics analyses were performed as previously described [34]. Differentially expressed genes (DEGs) were identified with the limma package, which implements an empirical Bayesian approach to estimate gene expression changes by using the moderated t-test [35]. |log FC| > 0.5 and *P* < 0.05 were considered cutoff criteria to screen for DEGs. Functional enrichment analyses of the detected DEGs were performed with the clusterProfiler package. GO and Kyoto Encyclopedia of Genes and Genomes (KEGG) terms were identified with a cutoff of *P* < 0.05. We also identified pathways that were upregulated or downregulated in NASH livers from 2 groups [36]. Gene sets for analysis were obtained from the MSigDB database; and enrichment analyses of DEGs were performed using Metascape. After finding the pathway we use the Cytoscape (Ver3.9.1) to search for differential genes. Finally, the mechanism diagram was rendered using Biorender software.

### Human liver specimens

Human liver specimens were taken from MASLD/MASH patients who had undergone surgical liver resection or liver biopsy; and healthy liver tissues were taken from the unaffected portion of the liver of patients having surgery to repair a hepatic hemangioma or trauma. All liver samples are obtained from Shanghai Public Health Clinical Center that approved by the ethics committees of Shanghai Public Health Clinical Center (2022-S104-01). Written informed permission was provided to all participants in accordance with the Helsinki Declaration. Table S3 displays data from laboratory and clinical studies.

### Histopathology and immunohistochemistry

For histopathological and immunohistochemical (IHC) assessment, paraffin-embedded formalin-fixed liver sections were stained with H&E, Masson’s trichrome, or sirius red according to standard protocols. Liver histology was blinded reviewed by two pathologists to conduct steatosis grading and NAFLD activity score (NAS) as previously described [32]. Additionally, liver fibrosis and fat content were also assessed blindly using ImageJ software based on Sirius red and H&E-stained sections, respectively.

IHC staining was done according to our earlier instructions [32]. In summary, sections with a thickness of 5 μm were dewaxed, hydrated, and then heated to induce antigen retrieval. After that, slides were blocked and incubated with primary antibodies against α-SMA, MCU, Cyto c, F4/80, anti-4-HNE, and anti-cleaved Caspase-3 over night at 4 °C. The primary and secondary antibodies used are listed in Supplemental Table S1. Sections were subsequently incubated with HRP-conjugated secondary antibodies and finally examined under a light microscope to detect. Immunostaining signaling was quantified at a predetermined threshold using free NIH ImageJ 1.49 for 6-9 slides/group, 5 fields per slide.

### Oil red O and BODIPY staining

Lipid accumulation was analyzed using an Oil red O (ORO) staining kit (ScienCell, California, USA, #0843) as described in the manufacturer’s protocol. AML12 or primary hepatocytes were fixed with 4% paraformaldehyde for 10 min. Then, cells were stained with freshly diluted 0.2% ORO solution in a 37 °C incubator for 10 min. Plates were counterstained with hematoxylin to stain cell nuclei. As for liver sections, ORO staining in sections from frozen liver tissue was conducted as described previously [30, 32]. The positive area was assessed from randomly selected 6 fields of ×200 magnification per slide and quantified with NIH ImageJ 1.49 software.

For BODIPY staining, the cells were treated and fixed the same with ORO staining. Then the fixed cells were stained with BODIPY 493/503 dye under RT for 10 min and protected from light, and the nuclei were stained with 4’,6-diamidino-2-phenylindole (DAPI). The stained lipid droplets were observed under a confocal microscope (FV3000, Olympus). The areas of the lipid droplets in different groups were analyzed using NIH Image J 1.49 software.

### Western blot

Western blot analysis was performed according to standard protocols as previously described [30, 32]. Cells and liver tissues homogenized in RIPA buffer were centrifugated at 4 °C, and protein concentrations were assessed by micro-BCA assay kit (Beyotime, Shanghai, China). Protein (10–20 µg) was dissolved by electrophoresis on 12.5% SDS-PAGE gels and transferred to polyvinylidene fluoride membranes. The membranes were blocked and incubated overnight at 4 °C with primary antibodies against MCU, β-Actin, Caspase-3, cleaved Caspase-3, α-SMA, CPT1A, AMPK, p-AMPK, YAP1, p-YAP1, LATS1/2, p-LATS1/2, MOB1, and p-MOB1. Followed by washing 3 times, the membranes were incubated with peroxidase-conjugated secondary antibodies under RT. The primary and secondary antibodies used are listed in Table S1. COX IV or β-actin was used as a loading control. Bands were visualized with ECL™ Western Blotting Detection Reagents (EpiZyme, Shanghai, China), and the optical density of the bands were determined using the NIH ImageJ software.

### RNA isolation and quantitative real-time PCR

Total RNA was extracted from cells or liver tissue using Trizol reagents (Takara) according to the manufacturer’s instructions. For qRT-PCR, cDNAs were reverse transcribed using GoScript™ Reverse Transcription Mix and Random Primers (Promega). Relative quantitative gene expression levels were measured by qRT-PCR using Promega SYBR Green mix kit on QuantStudio 5 System (Applied Biosystems, CA). β-actin was used as an internal control, and the relative expression of target genes was calculated using the 2^−ΔΔCT^ method. All primers used are listed in Table S4.

### TUNEL staining

Terminal deoxynucleotidyl transferase-mediated dUTP nick-end labeling (TUNEL) staining was utilized to identify apoptotic cells in liver sections and in vitro AML12 hepatocytes. Formalin-fixed and paraffin-embedded liver sections were stained using the DeadEnd™ Fluorometric TUNEL System (#G3250; Promega, Wisconsin, USA) following the manufacturer’s instructions. Olympus BX43 microscope was applied to view the slides after they had been counterstained with DAPI. Then recordings were made using an Olympus FV3000 confocal microscope.

### Flow cytometry analysis

Apoptosis/necrosis was performed using a FITC Annexin V Apoptosis Detection Kit I (#556547; Becton Dickinson Biosciences, USA) according to the manufacturer’s protocol. Briefly, AML12 cells were treated as indicated and harvested, collected, and resuspended in 100 µL of binding buffer and stained with annexin V FITC (5 µL) and propidium iodide (PI) staining solution (5 µL) for 20 min at RT in the dark. Samples were chilled before testing, and flow cytometry was employed to detect cellular apoptosis. Data were analyzed using the Flow Jo software (V10.8.1).

### Determination Caspase3/7 activity

Caspase-3/7 activities in cells were measured using the Caspase-3/7 Activity Apoptosis Assay Kit *Green Fluorescence (Sangon Biotech, Shanghai, China) according to the manufacturer’s protocol as described previously [32]. Briefly, cultured with or without OPA for 24 h, AML12 cells were lysed with buffer containing caspase substrate TF2-DEVD-FMK and incubated for 1 h at RT. Caspase3/7 activities were measured using FlexStation3 multifunctional microplate reader (Molecular Devices, California, USA) with excitation wavelength at 490 nm and emission wavelength at 525 nm.

### Assessment of hepatic oxidative stress and lipid peroxidation

As previously mentioned, the Lipid Peroxidation MDA Assay Kit was utilized in accordance with the manufacturer’s directions to measure the amount of lipid peroxidation in the mice’s livers. The manufacturer’s procedure was followed to evaluate the hepatic Catalase activity using the Catalase Assay Kit. The results were expressed as micromoles of decomposed H_2_O_2_ per minute per milligram of liver protein. By measuring the amounts of superoxide anion using DHE staining, as previously described, oxidative stress was identified in frozen and unfixed liver tissue slices. Summing up, cryosections of the livers from each mouse group were incubated for 30 min at 37 °C with 25 µM DHE in a light-protected, humidified chamber. After that, they were rinsed three times with PBS. DHE fluorescence was captured using an Olympus BX43 fluorescence microscope.

### Hydroxyproline content

Hydroxyproline content of the livers was measured using the hydroxyproline detection kit (#A030-2-1; Jiancheng, Nanjing, China) according to the manufacturer’s instructions. Briefly, liver tissue (40 µg) was hydrolyzed with 1 ml of hydrolysate, and bathed in water at 95 °C for 20 min. After cooling the tubes to RT in running water, we added 10 µL of pH indicator, solution A, and solution B to each tube sequentially to adjust the pH value to 6.0-6.8. The liquid in the tubes was then diluted to a volume of 10 mL. 4 mL of diluted hydrolysate was mixed with the required amount of activated carbon, and the mixture was then centrifuged at 3,500 rpm for 10 min to collect the supernatant. Sample and standard were used to measure the amount of hydroxyproline in 100 µL of supernatants at a wavelength of 550 nm. Using the formula, collagen levels were estimated from the product of hydroxyproline content (µg/g liver).

### Measurement of ALT and AST in serum

Blood was collected and stored at -80 °C until use. Serum aspartate aminotransferase (AST) and alanine aminotransferase (ALT) were evaluated with a commercial assay kit according to the manufacturer’s recommendations (Nanjing Jiancheng Biological Technology, Nanjing, China). Liver enzyme activities were shown in international unit per liter (U/L).

### Measurement of intracellular ROS and mitochondrial superoxide levels

Cellular ROS generation was measured using the ROS Assay Kit as previously described. Briefly, after being stained with 10µM DCFH-DA for 20 min at 37 °C, the incubated cells were washed and lysed in PBST. Finally, fluorescence was measured at 495 nm/530 nm (Ex/Em) under Olympus BX43 microscope or Flow cytometry (FACS Arial, Becton Dickinson). Staining for mitochondrial superoxide (miROS) was performed using the MitoSOX Red indicator following the manufacturer’s instructions as previously described.

### Transmission electron microscopy

For electron microscopy, Kreb’s solution and a 0.15 M cacodylate buffer containing 2.5% glutaraldehyde, 2% paraformaldehyde, and 2 mM CaCl_2_ were both injected into mice, followed by 5 min of heating to 37 °C. After perfusion, liver tissue was carefully removed and once again fixed for the night at 4 °C. The tissues were washed 3 times in 0.15 M cacodylate buffer, then post-fixed with 1% osmium tetroxide and 0.3% potassium ferrocyanide in 0.15 M cacodylate buffer with 2 mM CaCl_2_. Following washing 3 times in ddH_2_O, liver tissue was en bloc stained with 2% aqueous uranyl acetate overnight at 4 °C and subsequently dehydrated in a Spurr’s resin (30%, 50%, 70%, and 100%) mixed with 100% ethanol, embedded in fresh 100% Spurr’s resin in silicon molds, and polymerized at 60 °C for 48 h. Following polymerization, the resin blocks were faced, 70 nm ultrathin sections were cut on a Leica EM UC7 ultramicrotome (Leica-Microsystems, Vienna, Austria), picked up on copper formvar/carbon support film grids (PN FCF100H-Cu, Electron Microscopy Sciences, Hatfield, PA), post-stained with 2% uranyl acetate for 15 min, followed by Reynolds lead citrate and imaged on 5–6 photos per sample at a magnification of 2000 were taken.

### ATP measurement

Hepatic ATP content was measured using the ATP Luminescence Kit (#40210ES10; Yeasen Biotechnology, Shanghai, China) according to the manufacturer’s instructions [32]. Briefly, after homogenization in RIPA lysis buffer, 20 mg of liver tissue was used. For intracellular assessment, 5 × 10^5^ cells/well were washed and then lysed in 100 µL buffer after treatment with OPA or vehicle. The lysate was finally centrifuged at 12,000×g for 3 min to collect the cell supernatant. Samples were added to a 96-well plate and then incubated with 100 µL of the test working fluid at RT in the dark for 2 s. Absorbance was read the Luminescene using FlexStation3 multifunctional microplate reader (Molecular Devices). ATP content was calculated based on a standard curve generated at the same time.

### Measurement of mitochondrial membrane potential (MMP)

The MMP was determined using the JC-1 Mitochondrial Membrane Potential Assay Kit (#10009172; Cayman, Michigan, USA) according to the manufacturer’s instructions [32]. Briefly, AML12 cells were seeded in a 12-well Glass Bottom Plate (Cellvis) and treated respectively. After being washed with PBS, cells were incubated with JC-1 for 30 min at 37 °C. Nuclei were counterstained with Hoechst. In a nutshell, Alexa Fluor 594 channel and Alexa Fluor 488 channel were adopted, respectively, for the detection of JC-1 in its J-aggregate form (red fluorescence) and monomeric form (green fluorescence). The ratio between red and green fluorescence was calculated to indicate the MMP.

### Measurement of mitochondrial Ca^2+^ concentration

Mitochondrial Ca^2+^ concentration was assessed as described previously [24]. Briefly, AML12 hepatocytes cultured in a 12-well Glass Bottom Plate (Cellvis) or confocal dishes (Biosharp Life Sciences) were treated separately. After being washed with PBS, cells were loaded with Rhod-2 AM (5 µM) for 30 min at 37 °C. The fluorescence of cells was observed under Opera Phoenix High-Content Screening System (PerkinElmer, CT) with Alexa Fluor 488 channel or a confocal microscope (Olympus FV3000) with Alexa Fluor 594 channel. Data were analyzed using ImageJ software.

### Measurement of mitochondrial oxygen consumption rate

Following the manufacturer’s instructions, liver mitochondria were separated from the appropriate animals (five mice per group) at the end of eight weeks using a tissue mitochondria isolation kit (Beyotime Institute Biotechnology) [32]. In a nutshell, 50 mg of liver samples were diced and homogenized (1:10 w/v) in cold mitogen buffer. After that, portions of the plasma membrane and nuclei were spun down by centrifuging the tissue homogenate. To get rid of impurities, the supernatant was filtered and centrifuged. In order to extract the mitochondria, we also extracted the cytoplasmic proteins from the supernatant. Next, the pellet was immersed in 100 µl of mitochondrial buffer.

Mitochondrial function was measured by oxygen consumption rate (OCR) using Seahorse XF96 Analyzer (Agilent Technologies, California, USA) as previously described [37]. Briefly, hepatic mitochondria were incubated in a mitochondrial assay solution. Substrate stocks containing 0.5 M succinic acid, 0.5 M malic acid, 0.5 M glutamic acid, 0.5 M pyruvic acid, and 1.0 M ADP, and adjusted to pH 7.2. Respiration reagent stocks were used: FCCP (10 mM), oligomycin (5 mg/mL), rotenone (2 mM), and antimycin A (40 mM). 2 µg mouse liver mitochondria work well in the Agilent Seahorse XF96 begin in a coupled state with substrate present. ADP, oligomycin, FCCP, Rotenone, and Antimycin were sequential additions to the reaction system. State 3 initiated with ADP, State 4 induced with the addition of oligomycin (State 4o), and FCCP-induced maximal uncoupler-stimulated respiration (State 3u) were sequentially measured, allowing respiratory control ratios. Respiratory control ratio (RCR) was defined as the ratio of state 3 respiration to state 4o. Data were collected using Agilent Seahorse Wave 2.6.1 Desktop software and analyzed by GraphPad Prism version 9.

### Statistical analysis

All data are presented as means ± standard deviations (SD), and the data have passed the normality and equal distribution test. Statistically significant differences between two independent groups were made by unpaired two-tailed Student’s *t*-test. Comparisons among multiple groups were performed by one-way ANOVA with Tukey’s *post hoc* honest significant difference test or two-way ANOVA followed by Tukey’s or Sidak’s multiple comparisons test. Statistical analyses were performed using GraphPad Prism 9.0 (San Diego, CA, USA). For all comparisons, significance is indicated as *P < 0.05, **P < 0.01, ***P < 0.001, and ****P < 0.0001; ns indicates not significant.

## Results

### Hepatic MCU expression is up-regulated in patients with MASLD and MASH

**Fig. 1:**
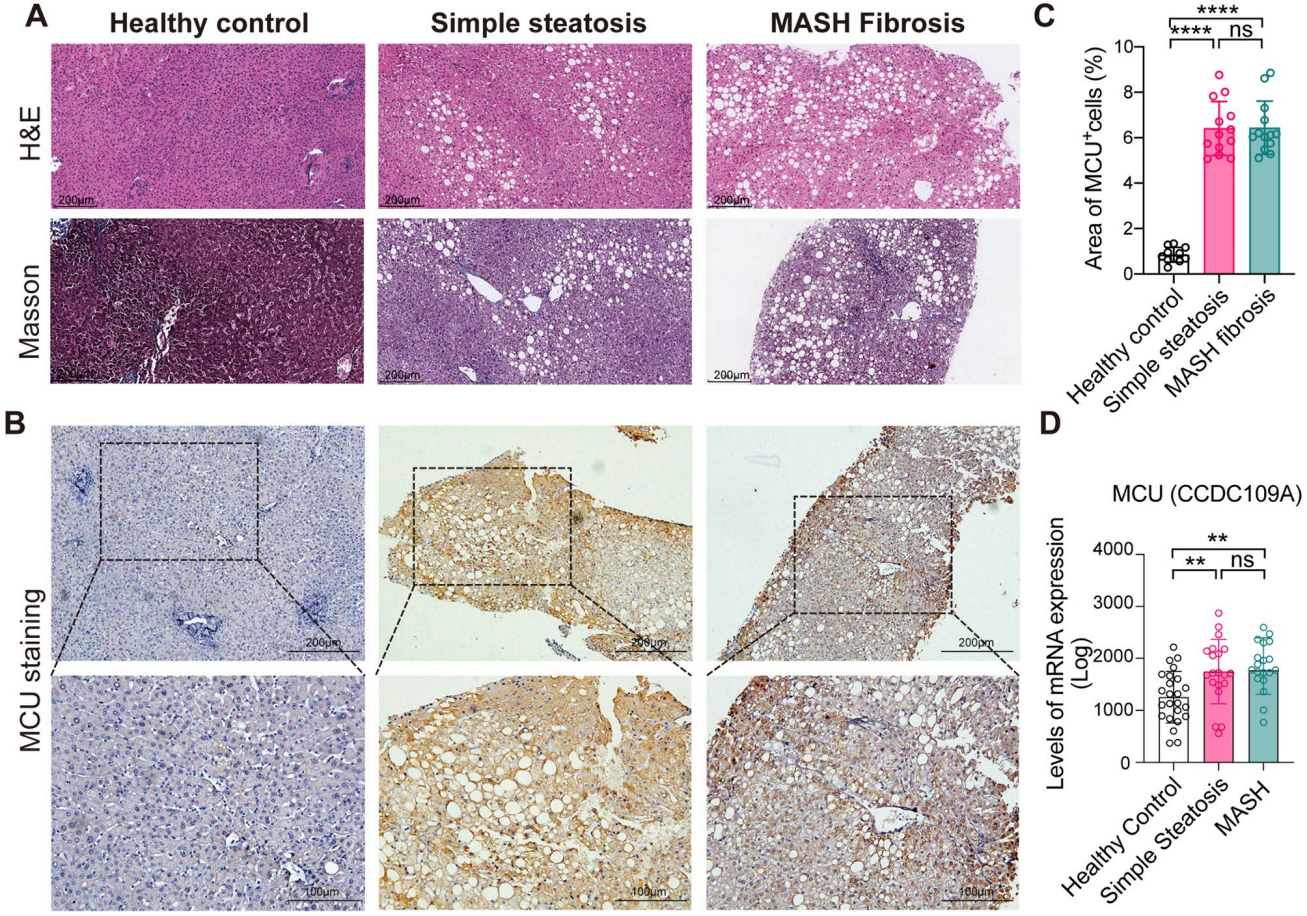
Hepatic MCU expression is upregulated in patients with MASLD and MASH. **A** Representative H&E and Masson’s trichrome staining in liver sections (scale bars: 200 μm). **B** Representative MCU immunostaining in liver sections from our cohort of MASLD/MASH patients. Bottom row (scale bars: 100 μm) contains images enlarged from the boxed area in the corresponding panel in the top row (scale bars: 200 μm). **C** Areas of MCU^+^ cells (%) per field (*n* = 11–13/group) were analyzed by ImageJ. **D** Database-based gene expression analysis was conducted using public data sets obtained from GEO at site the NCBI (http://www.ncbi.nlm.nih.gov/geo/). The data were processed as median-normalized signal intensity values. Healthy control (*n* = 24), simple steatosis (*n* = 20) and MASH (*n* = 19) for GEO: GSE89632

To determine whether MCU contributes to human MASLD/MASH, we used IHC to assess its expression in liver biopsies from patients with MASLD, which were diagnosed blind by two pathologists into the following categories: normal, simple steatosis, and MASH with fibrosis. H&E and Masson’s trichrome staining were conducted to indicate the extent of liver steatosis and fibrosis (Fig. 1A). MCU expression was only weak in healthy livers and strikingly enhanced in MASLD/MASH livers; and MCU largely localized in hepatocytes of MASLD livers, particularly at the portal tracts and fibrous septa (Fig. 1B). These observations were consistent with the increased area of MCU-positive cells in liver sections from simple steatosis and MASH. But no significant difference in MCU expression between simple steatosis and MASH group was observed (Fig. 1C). In line with our findings, analysis of the human GenBank data (GEO: GSE8932) consisting of 39 MASLD patients shows that a higher expression of liver MCU mRNA in MASLD patients than that in healthy controls (Fig. 1D). Thus, these data in humans strongly suggest that MCU might mediate the pathology of MASLD/MASH.

### Hepatic MCU expression is up-regulated in MASH mice

**Fig. 2:**
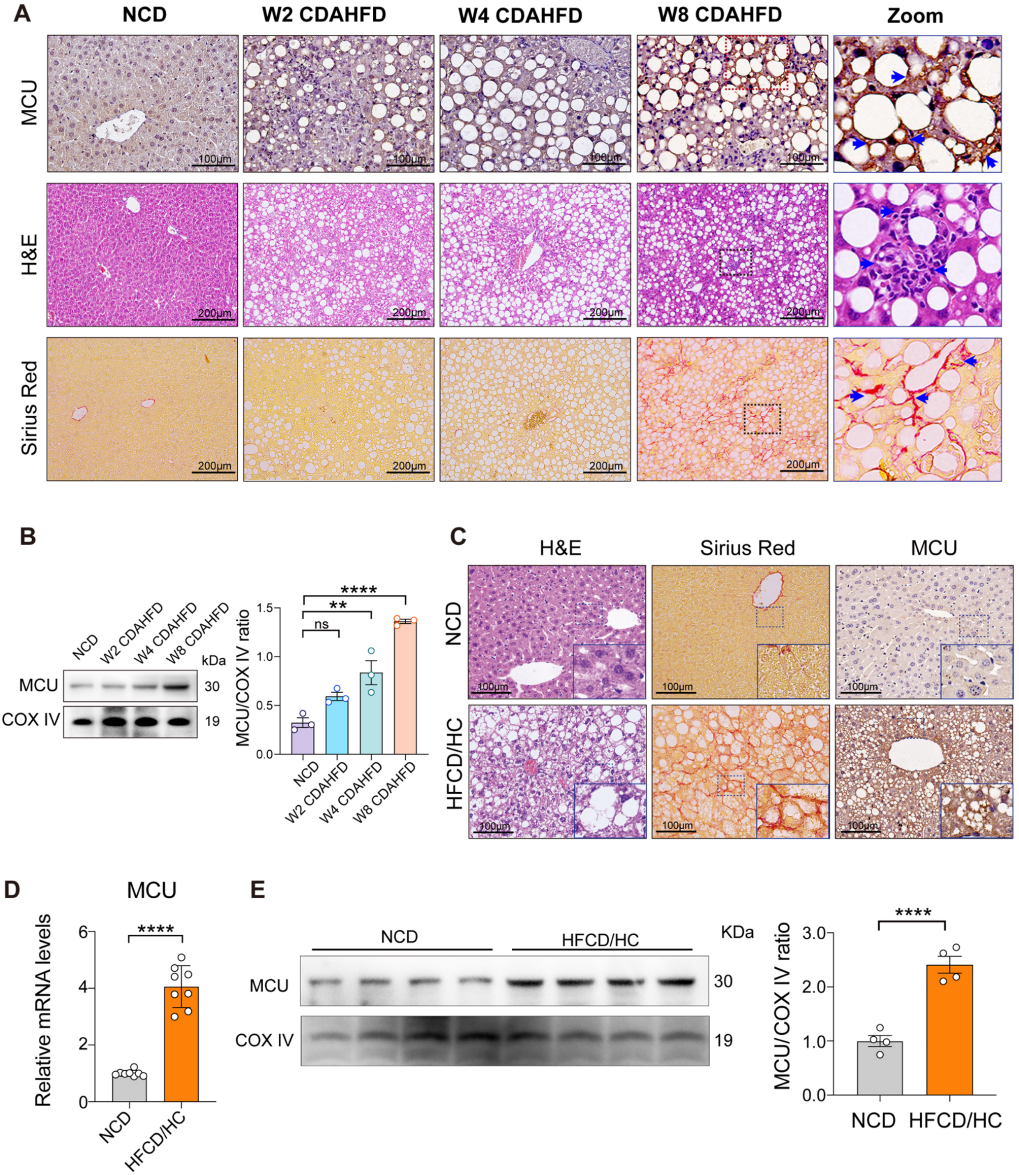
Hepatic MCU expression is upregulated in MASH mice. **A** Histology of liver sections from NCD or CDAHFD-fed mice stained for H&E, Sirius red as well as MCU immunostaining. Insert showing the typical MCU-positive cells, lobular inflammation, and pericellular fibrosis (arrows). Scale bars: 100 μm (top panel) and 200 μm (bottom two panels). **B** Western blot analyses for MCU expression in the livers from indicated mice; results were normalized relative to expression of COX IV. **C** Representative H&E, Sirius red, and MCU immunohistochemical staining in liver sections. Mice fed with a HFCD/HC diet or a NCD diet for 16 weeks. Insert showing the typical hepatocyte ballooning, pericellular fibrosis, and MCU-positive cells. Scale bars: 100 μm. **D** Relative MCU mRNA level in livers was assessed using qRT-PCR. Results were normalized to β-actin mRNA and expressed as fold change. *n* = 8 mice/group. **E** Western blot analyses for MCU expression in the liver from NCD-fed mice or HFCD/HC-fed mice; results were normalized relative to expression of COX IV. Data are mean ± SD. One-way ANOVA with Tukey’s post-test (**B**) and Two-tailed unpaired Student’s *t*-test (**D**, **E**). *P < 0.05, **p < 0.01, ***P < 0.001, and ****p < 0.0001; ns indicates not significant

Next, we used IHC to assess liver MCU expression in a murine MASH model induced by CDAHFD-feeding. Hepatic MCU expression gradually enhanced as MASLD progression and was the most pronounced in MASH induced by CDAHFD-feeding 8 weeks, and hepatic MCU was mostly localized in hepatocytes (Fig. 2A). Moreover, western blot analysis further verified the enhanced expression of MCU in the MASLD livers as compared with the normal livers (Fig. 2B).

Likewise, MCU expression was also assessed in another established model of mice fed an HFCD/HC diet for 16 weeks [31]. As expected, mice fed HFCD/HC displayed several key features of MASH pathology, including steatosis, lobular inflammation, and fibrosis as evidenced by H&E and Sirius red staining in liver sections. Similarly, MCU was strikingly expressed in steatotic hepatocytes (Fig. 2C); and expression of MCU mRNA and protein significantly upregulated in MASH livers compared with the livers from NCD-fed mice (Fig. 2D and E). Taken together, our data demonstrate that hepatic MCU is involved in the pathogenesis of MASH and fibrosis.

### MCU deficiency attenuates mitochondrial Ca^2+^ uptake, oxidative stress, and mitochondrial dysfunction in AML12 hepatocytes under lipotoxicity

**Fig. 3:**
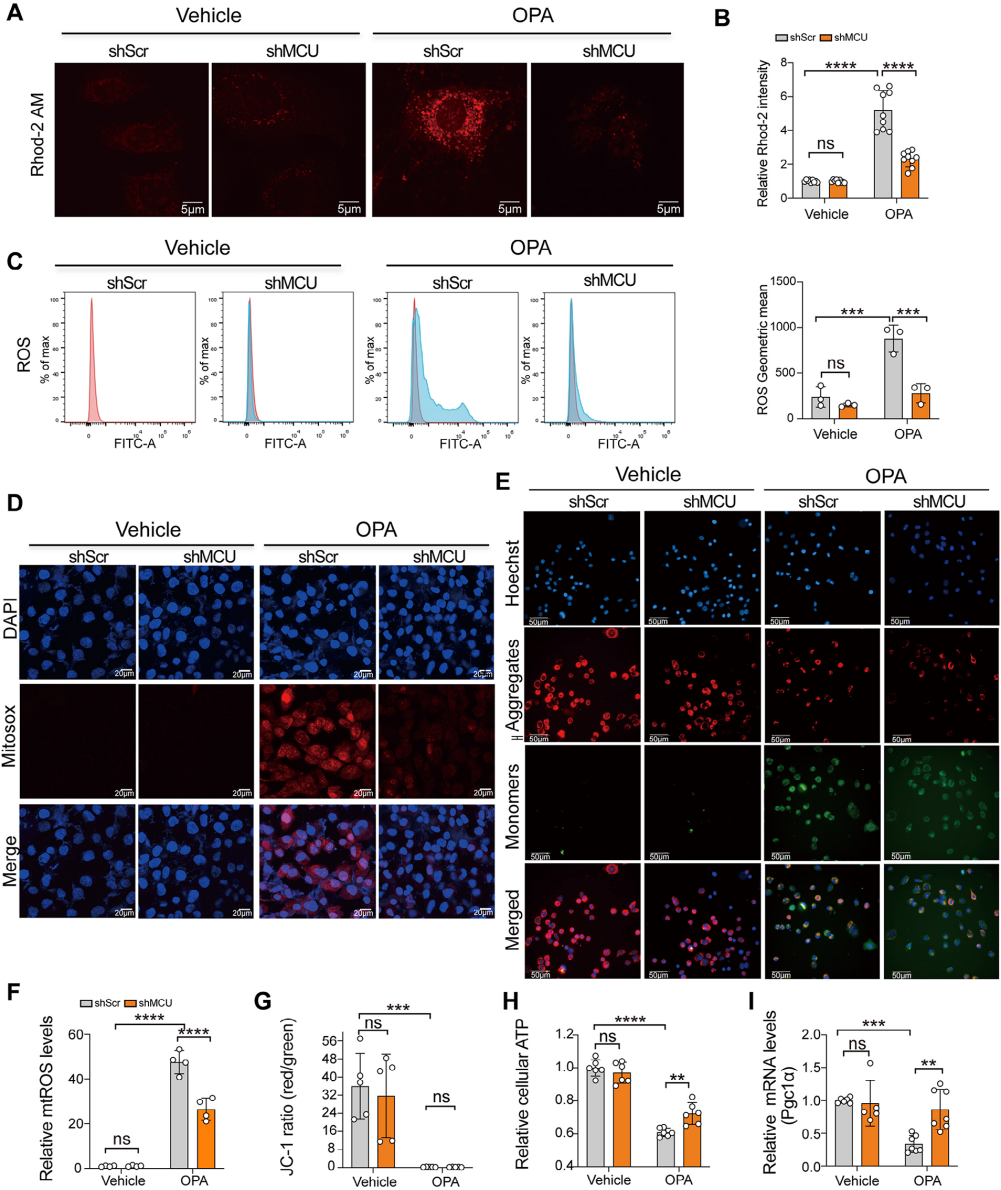
MCU deficiency attenuates mitochondrial Ca^2+^ uptake, oxidative stress, and mitochondrial dysfunction in AML12 hepatocytes under lipotoxicity. **A** Representative images of mitochondrial Ca^2+^ fluorescence (Rhod-2 Ca^2+^ indicators) in AML12 hepatocytes. Scale bars: 5 μm. **B** Rhod-2 Ca^2+^ quantified using ImageJ software (*n* = 9 fields). **C** ROS levels assessed by flow cytometry using H_2_DCFDA in AML12 hepatocytes and signal was measured by flow cytometry. **D** Representative image of MitoSOX in AML12 hepatocytes. Scale bars: 20 μm. **E** Mitochondrial membrane potential (MMP, Δψm) in AML12 hepatocytes was detected by JC-1 dye staining and visualized with a fluorescence microscope (×400). Scale bars: 50 μm. **F** Quantification of mitochondrial ROS (miROS) based on MitoSOX Red fluorescence (**D**). The data were pooled from three independent experiments. **G** Quantification of JC-1 fluorescence in AML12 hepatocytes. The data were pooled from three independent experiments. **H** Relative intracellular ATP content. **I** qRT-PCR analysis of *Pgc1α* mRNA expression. Results were normalized to β-actin mRNA and expressed as fold change compared to control group. Data are mean ± SD. Two-way ANOVA with Tukey’s test (**B**, **C**, **F**, **G**, **H**) and two-way ANOVA with Sidak’s test (**I**). **P < 0.01, ***P < 0.001, and ****P < 0.0001; ns indicates not significant

To validate whether MCU-mediated Ca^2+^ signaling plays a key role in lipid metabolism and lipotoxicity, AML12 hepatocytes were transfected with shMCU or shScr. Following stable transfection, the cells were treated by OPA for 24 h, and then the silencing efficiency was confirmed by qRT-PCR and western blot respectively (Additional file 1: Fig. S2G and H). We evaluated the Ca^2+^ concentrations in mitochondria (mCa^2+^) in AML12 hepatocytes using the Rhod-2 AM fluorescence and revealed increased levels of mCa^2+^ in AML12 hepatocytes as confirmed by Rhod-2 fluorescence intensity. However, silencing of MCU blunted the increased levels of mCa^2+^ in OPA-treated AML12 hepatocytes (Fig. 3A and B).

Next, knockdown of MCU in hepatocytes was found to attenuate oxidative stress under OPA challenge, as reflected by decreased intracellular ROS levels (Fig. 3C) and DHE fluorescence (Additional file 1: Fig. S3C and D). Moreover, OPA-treated AML12 hepatocytes enhanced the production of mtROS as measured by MitoSOX fluorescence; however, MCU-deficient abrogated the increased mtROS in OPA-treated AML12 hepatocytes (Fig. 3D and F). To further determine whether oxidative stress by lipotoxicity was associated with loss of the mitochondrial membrane potential (MMP, ∆ψ_m_), AML12 hepatocytes were incubated with OPA followed by staining with JC-1 probe. Obviously, OPA lipotoxicity elicited MMP in AML12 hepatocytes; however, there was no changes in MMP between MCU-deficient AML12 hepatocytes and control cells under OPA overload (Fig. 3E and G). Moreover, we noted that OPA-induced AML12 hepatocytes robustly reduced ATP production, but this reduction was inhibited by MCU knockdown (Fig. 3H). Additionally, given peroxisome proliferator-activated receptor-γ coactivator-1α (PGC-1α) is involved in mitochondrial biogenesis and fatty acid oxidative [38], we examined the expression of *Pgc1α* mRNA in OPA-treated AML12 hepatocytes by qRT-PCR and found an enhanced level of *Pgc1*α expression in MCU-silenced AML12 hepatocytes under lipotoxic stress (Fig. 3I).

Taken together, our results reveal that MCU deficiency in AML12 hepatocytes reduced mitochondrial Ca^2+^ uptake, attenuated hepatic oxidative stress, and mitochondrial dysfunction in lipotoxicity.

### Knockdown of hepatic MCU limits liver injury, steatosis, and inflammation in MASH mice

**Fig. 4:**
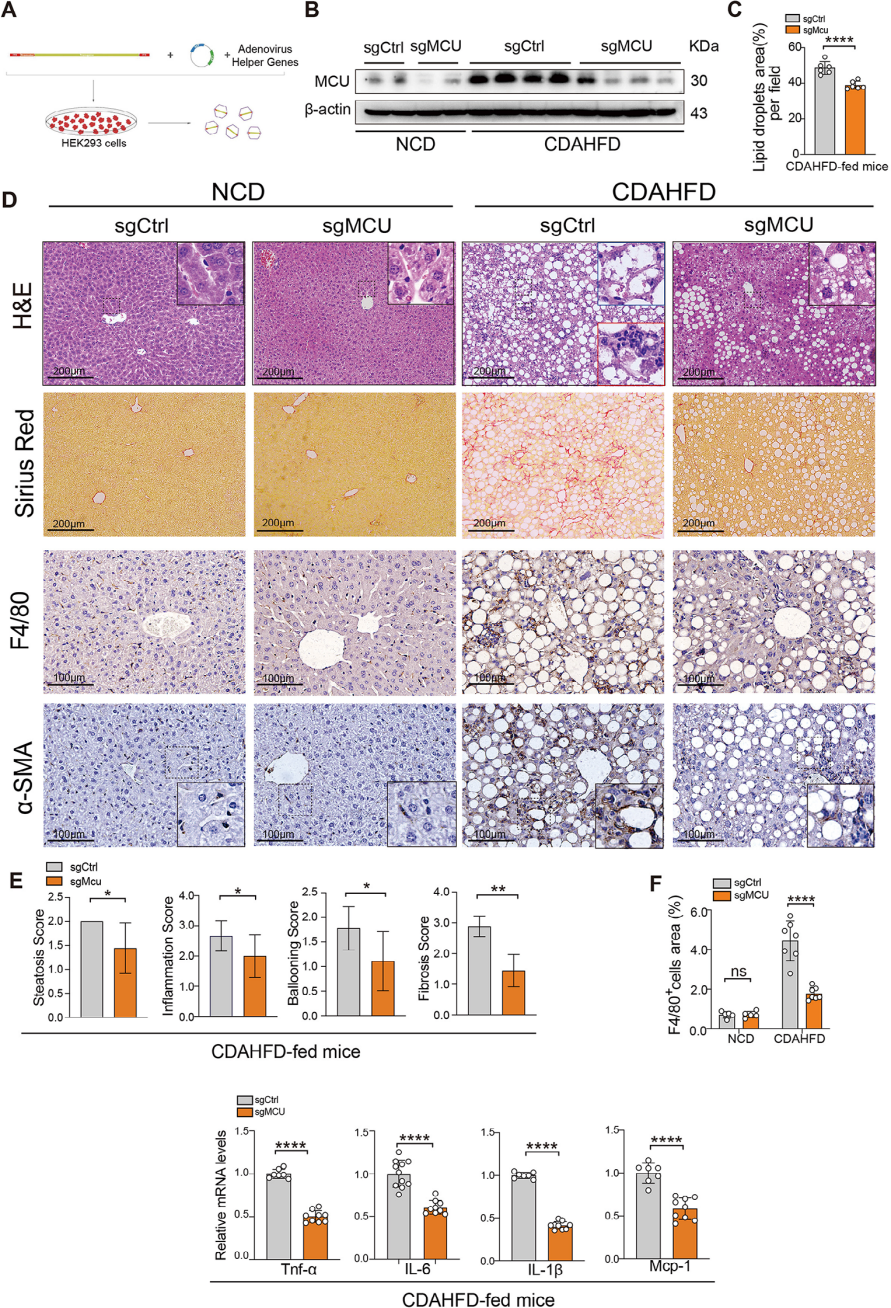
Knockdown of hepatic MCU limits liver injury, steatosis, and inflammation in MASH mice. **A** Flow chart illustrates the preparation of AAV8-mediated signal guide RNA (sgRNA). AAV8 helper plasmid, adenovirus helper genes, and sgRNA adenoviral vector were co-transfected into HEK293 cells to induce sgRNA expression for MCU deletions. **B** Western blots analysis of MCU in livers from indicated mice; COX IV served as a loading protein. **C** Representative H&E and F4/80 staining in liver sections form indicated mice. Insert show the typical morphology of ballooning hepatocytes (blue box) and lymphocytic infiltrates (red box). Scale bars: 200 μm (upper 2 panel) and 100 μm (lower 2 panels). **D** Hepatic lipid droplets area was quantified based on H&E staining (*n* = 6 mice/group). **E** Histological analysis using NAFLD activity score, and hepatocyte ballooning, steatosis, and inflammation as scored quantitatively from H&E-stained sections (*n* = 9 mice/group). **F** Quantification of F4/80^+^ cells per high-power field in liver sections (*n* = 5–7 mice/group). **G** Relative mRNA expression of *Tnfα*, *Il6*, *Il1β*, and *Mcp1* in livers assessed by qRT-PCR; and the results were shown as fold change compared with NCD control and normalized to β-actin mRNA. Data are mean ± SD. Two-tailed unpaired Student’s t-test (**D**, **E**, **G**) and two-way ANOVA with Sidak’s test (**F**). **P < 0.01, ***P < 0.001, and ****P < 0.0001; ns indicates not significant

To characterize the roles of MCU in the pathogenesis of MASH, we used CRISPR/Cas9 gene targeting to inactivate MCU specifically in the liver; and AAV8-mediated sgRNA targeting MCU exons (sgMCU) was injected via tail vein to mice (Fig. 4A and Additional file 1: Fig. S1). Efficiency of MCU knockdown in the liver was verified by western blot (Fig. 4B). Liver sections in CDAHFD-fed mice display histological alterations similar to the main pathologic feature of human MASH, indicating steatosis, hepatocyte ballooning, inflammation, and fibrosis. However, these morphological alterations are remarkably alleviated in MCU-deficient MASH mice (Fig. 4C). These observations are consistent with the lower NAS grading and individual scores for inflammation, steatosis, hepatocyte ballooning, and fibrosis in MCU-deficient MASH mice (Fig. 4E).

Next, liver macrophage infiltration is assessed by IHC for F4/80. As expected, WT mice fed a CDAHFD lead to an increased macrophage infiltration into livers, however, MCU-deficient MASH mice display reduced infiltration of F4/80^+^ cells in livers (Fig. 4C and F). Furthermore, we evaluated the expression of proinflammatory marker genes, such as *Tnfα*, *Il6*, *Il1β*, and monocyte chemoattractant protein-1 (*Mcp1*), which are significantly lower in MASH livers from MCU knockdown mice than those from control mice (Fig. 4G). Additionally, under OPA challenge, MCU silencing AML12 hepatocytes abrogate gene expression of proinflammatory factors such as *Il1β* and *Il6* (Additional file 1: Fig. S3A and B).

There are significant differences in body weight (BW), liver weight (LW), and liver/body weight ratio between CDAHFD-fed mice and NCD-fed mice. However, LW gain and ratios of liver/body weight are lower in MCU-deficient MASH mice than in WT MASH mice; no differences in BW gain were detected between MASH groups. CDAHFD-fed mice induced liver injury as confirmed by the levels of serum ALT and AST, but attenuating in MCU-deficient mice; MCU-deficient mice or control mice with chow diet had no damage to the liver (Table S3).

Taken together, these findings indicate that knockdown of hepatic MCU protected against liver injury, steatosis, and inflammation in MASH mice.

### Knockdown of hepatic MCU mitigates liver fibrosis and inhibits HSCs activation in diet-induced MASH mice

**Fig. 5:**
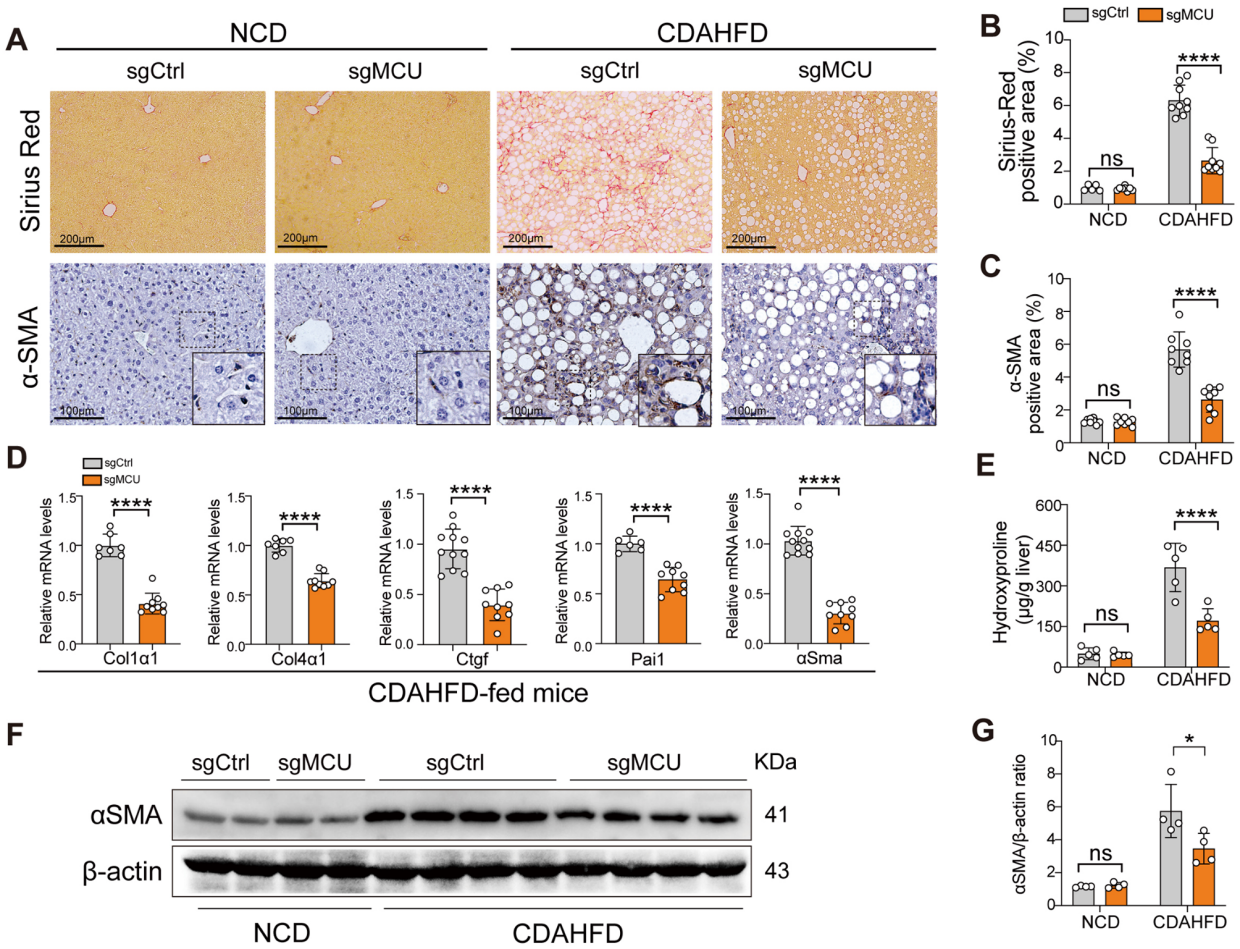
Knockdown of hepatic MCU mitigates liver fibrosis and inhibits HSCs activation in a diet-induced MASH mice. **A** Representative Sirius Red and αSMA staining in liver sections form indicated mice. Insert show the typical α-SMA^+^ cells (black box). Scale bars: 200 μm (upper 2 panel) and 100 μm (lower panels). **B** Quantitative analysis of sirius red positive area (*n* = 5–9 mice/group). **C** Quantification of the area of α-SMA^+^ cells (*n* = 8 mice/group). **D** Relative mRNA expression of fibrotic markers (*Col1α1*, *Col4α1*, *Ctgf*, *Pai1*, and *αSma*) levels in livers from indicated mice assessed using qRT-PCR. Results were normalized to β-actin mRNA and expressed as fold change. **E** Hepatic hydroxyproline content (µg/g liver) (*n* = 5 mice). **F** Representative western blots showing α-SMA; β-actin as loading control. **G** Densitometric analyses of blot for α-SMA/β-actin. Data are mean ± SD. Two-tailed unpaired Student’s *t*-test (**D**), two-way ANOVA with Tukey’s test (**C**, **E**, **G**), and two-way ANOVA with Sidak’s test (**B**). *P < 0.05, and ****P < 0.0001; ns indicates not significant

MCU-deficient mice fed with a CDAHFD diet exhibited markedly reduction in the accumulation of collagen and fibrosis (Fig. 5A and B). This observation was supported by the decreased mean fibrosis score based on H&E staining (Fig. 4E). Accordingly, MCU-deficient MASH mice led to a 53.44% reduction in content of hepatic hydroxyproline relative to control MASH mice (Fig. 5E). We next assessed the expression of fibrotic marker genes, such as collagen type 1 alpha1 (*Col1α1*), *Col4α1*, connective tissue growth factor (*Ctgf*), plasminogen activator inhibitor-1 (*Pai1*), and *αSma*, which were significantly lower in MCU-deficient MASH livers than these in the WT MASH livers (Fig. 5D).

Persistent inflammation of the liver can activate hepatic stellate cells (HSCs), which are the main producers of excessive extracellular matrix (ECM) and triggers of fibrogenesis, thus represents a more progressive course of disease and determines the mortality [1, 4]. We assessed the activated HSC using IHC staining for α-SMA in liver sections from each group mice. The result of our immunostaining exhibited a markedly increased number of activated HSCs in MASH livers, while MCU-deficient mice abrogated this activation of HSCs (Fig. 5A and C). In line with this observation, the gene and protein expression of α-SMA in livers were reduced in MCU-deficiency mice as compared with WT mice (Fig. 5D, F). Collectively, these results indicate that MCU-deficient mice strikingly limited MASH-associated liver fibrosis.

### Knockdown of MCU reduces lipid accumulation in hepatocytes during MASLD development both in mice and in cellular models

**Fig. 6:**
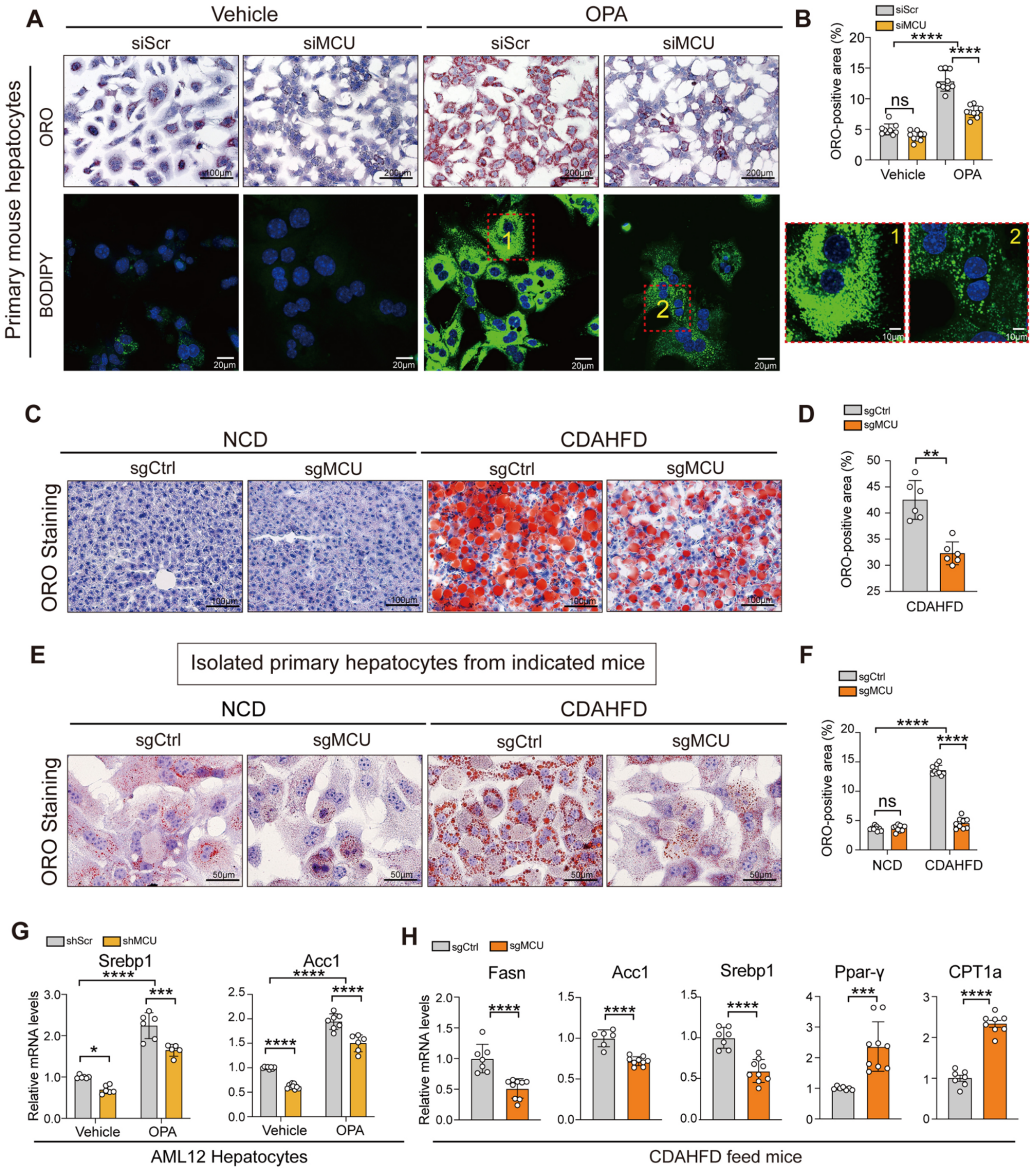
Knockdown of MCU reduces lipid accumulation in hepatocytes during MASLD development both in mice and in cellular models. **A** Representative microphotographs of ORO and BODIPY staining of primary mouse hepatocytes. Scale bars: 200 μm (top panel) and 20 μm (bottom panel). Boxed areas in fluorescence images are magnified in right panels. **B** Lipid accumulation in cells was quantified based on ORO-staining using the software NIH ImageJ (*n* = 4 biological replicates). **C** Liver sections were stained with ORO to reveal lipid droplets (scar bars: 100 μm). **D** Quantification of the ORO^+^ area in livers (*n* = 6 mice/group). **E** Lipid droplets accumulation in isolated primary hepatocytes from indicated mice revealed by ORO-staining. Scale bar: 50 μm. **F** Lipid droplets accumulation in hepatocytes (**E**) were quantified based on ORO-staining (*n* = 9 fields/group). **G** Relative mRNA expression of *Srebp1c* and *Acc1* in AML12 cells on response to OPA or vehicle assessed by qRT-RNA. Results were normalized to β-actin mRNA and expressed as fold change compared to control group. **H** Relative mRNA expression of *Fasn*, *Acc1*, *Srebp1c*, *Pparγ and Cpt1α* in mouse livers assessed by qRT-PCR. Results were normalized to β-actin mRNA and expressed as fold change. Data are mean ± SD. Two-tailed unpaired Student’s *t*-test (**D**, **H**) and two-way ANOVA with Turkey’s test (**B**, **F**, **G**). *P < 0.05, ** *P* < 0.01, *** *P* < 0.001, and ****P < 0.0001; ns indicates not significant

To dissect the role of MCU in hepatosteatosis, primary mouse hepatocytes were transfected with siMCU or siScr and were following treated with OPA or vehicle for 24 h. Strikingly, lipotoxic OPA overload led to profound lipid droplets in hepatocytes, as confirmed by ORO and BODIPY staining, but MCU silencing robustly blocked the deposition of lipid droplets in hepatocytes challenged by OPA (Fig. 6A, B). Next, we analyzed the transcript levels of the key enzymes mediating in the regulation lipogenesis and mitochondrial β-oxidation, such as sterol-regulating element-binding protein-1c (*Srebp1c*), acetyl-CoA carboxylase 1 (*Acc1*), and carnitine palmitoyltransferase 1α (*Cpt1α*). Remarkably, MCU knockdown led to reduced levels of *Srebp1c* and *Acc1* mRNA, and an increase in levels of *CPT1α* mRNA in OPA-treated AML12 hepatocytes (Fig. 6G). Thus, MCU promotes fat accumulation during hepatosteatosis in vitro.

Similarly, MCU-deficient mice fed with the MASH diet exhibited significantly reduced fat accumulation in hepatocytes in livers as verified by H&E and ORO staining (Fig. 6C and D), accompanied by smaller and less abundant in lipid droplets (Fig. 6C). At the end of 8 weeks, primary hepatocytes were isolated from mice fed with CDAHFD diet or NCD diets; followed ORO staining, we found lipid droplets in hepatocytes from CDAHFD-feeding mice strikingly increased compared with those from chow-diet mice, while hepatocytes from MCU-deficient MASH livers displayed a lower in lipid droplets deposition and ORO^+^ area (Fig. 6E and F). Supporting these results, MCU-deficient MASH mice limited the expression of *Fasn*, *Acc1*, and *Srebp1c*, and increased *PPARγ* mRNA (Fig. 6H). Accordingly, RNA-Seq datasets identified 50 significantly differentially expressed genes (DEGs) mediating in hepatic lipid metabolism between MCU-deficient MASH mice and WT MASH mice (Additional file 1: Fig. S4).

Collectively, our data indicate that MCU play a key role in liver metabolism and lipid accumulation in hepatocytes during MASLD development.

### MCU drives hepatocytes apoptotic death in MASH pathogenesis both in mice and in cellular models

**Fig. 7:**
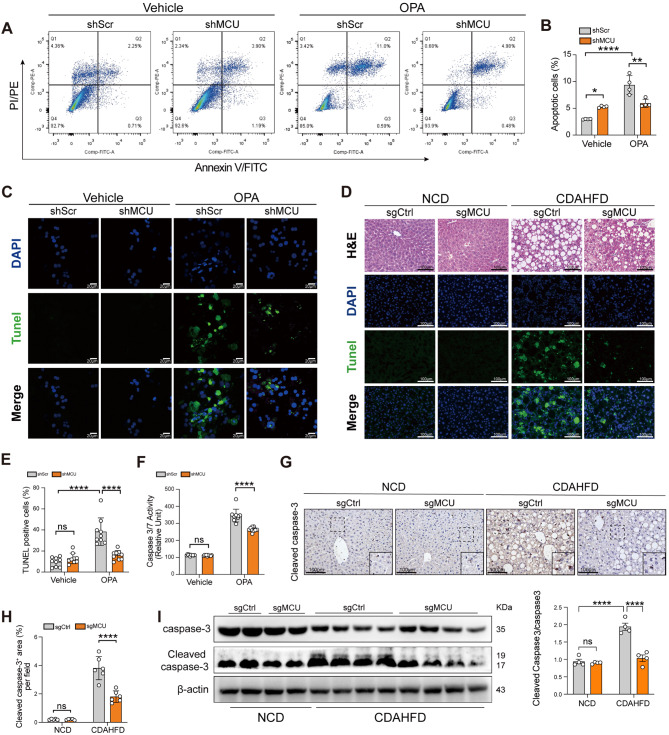
MCU drivers hepatocytes apoptotic death in MASH pathogenesis both in mice and in cellular models. **A-C** AML12 hepatocytes stable transfected with shMCU or shScr, then the transfected cells were treated with OPA or vehicle for 24 h. Cell apoptosis was detected using a Annexin V-FITC Apoptosis Detection Kit. Double-staining cells AML12 hepatocytes were defined as apoptotic cells (**A**) and quantification of apoptotic hepatocytes (**B**; *n* = 4). Representative TUNEL staining in hepatocytes from indicated group (**C**). Scale bar: 20 μm. **D** Representative H&E, TUNEL staining in liver sections. Scale bars: 100 μm. **E** Quantification of TUNEL-positive area in AML12 hepatocytes based on (**C**). **F** Caspase 3/7 activity in AML12 hepatocytes treated for 24 h either with vehicle or OPA. **G** Representative images of cleaved caspase-3 staining in liver sections. Scale bars: 100 μm. **H** Quantification of cleaved caspase-3^+^ area in liver sections (*n* = 5–6 mice/group). **I** Left: Western blots for cellular extracts full caspase-3 and cleaved caspase-3 and analyses of blots; β-actin served as a loading protein; Right: Densitometric analyses of blot for cleaved caspase-3/full caspase-3. Data are mean ± SD. Two-way ANOVA with Tukey’s test (**B**, **E**, **F**, **I**), and two-way ANOVA with Sidak’s test (**H**). *P < 0.05, **P < 0.01, and ****P < 0.0001; ns indicates not significant

To further understand the role of MCU in hepatocyte death during lipotoxicity, apoptotic death cells were assessed. Using flow cytometry followed staining with Annexin V/FITC, we found that MCU knockdown in hepatocytes limited the number of apoptotic cells induced by OPA (Fig. 7A and B). Additionally, MCU-deficient hepatocytes protected from OPA-induced apoptosis as reflected by TUNEL staining (Fig. 7C and E), the activity of caspase3/7 (Fig. 7F), and the levels of cleaved caspase-3 (Additional file 1: Fig. S5B). In mice test, TUNEL staining in liver sections revealed a remarkably increased apoptosis in MASH livers than in NCD-fed livers; however, MCU-deficient MASH livers displayed fewer apoptotic hepatocytes compared with these in WT MASH livers (Fig. 7D and Additional file 1: Fig. S5A). In agreement with these observations, MCU knockdown mice fed CDAHFD led to reduced apoptotic hepatocyte death as also confirmed by reduced cleaved caspase-3 levels (Fig. 7G-I). Altogether, our results indicate targeting MCU inhibited hepatocyte death in MASH mice and in vitro model mimic hepatosteatosis.

### Knockdown of hepatic MCU reduces hepatic oxidative stress and improves mitochondrial dysfunction in experimental MASH

**Fig. 8:**
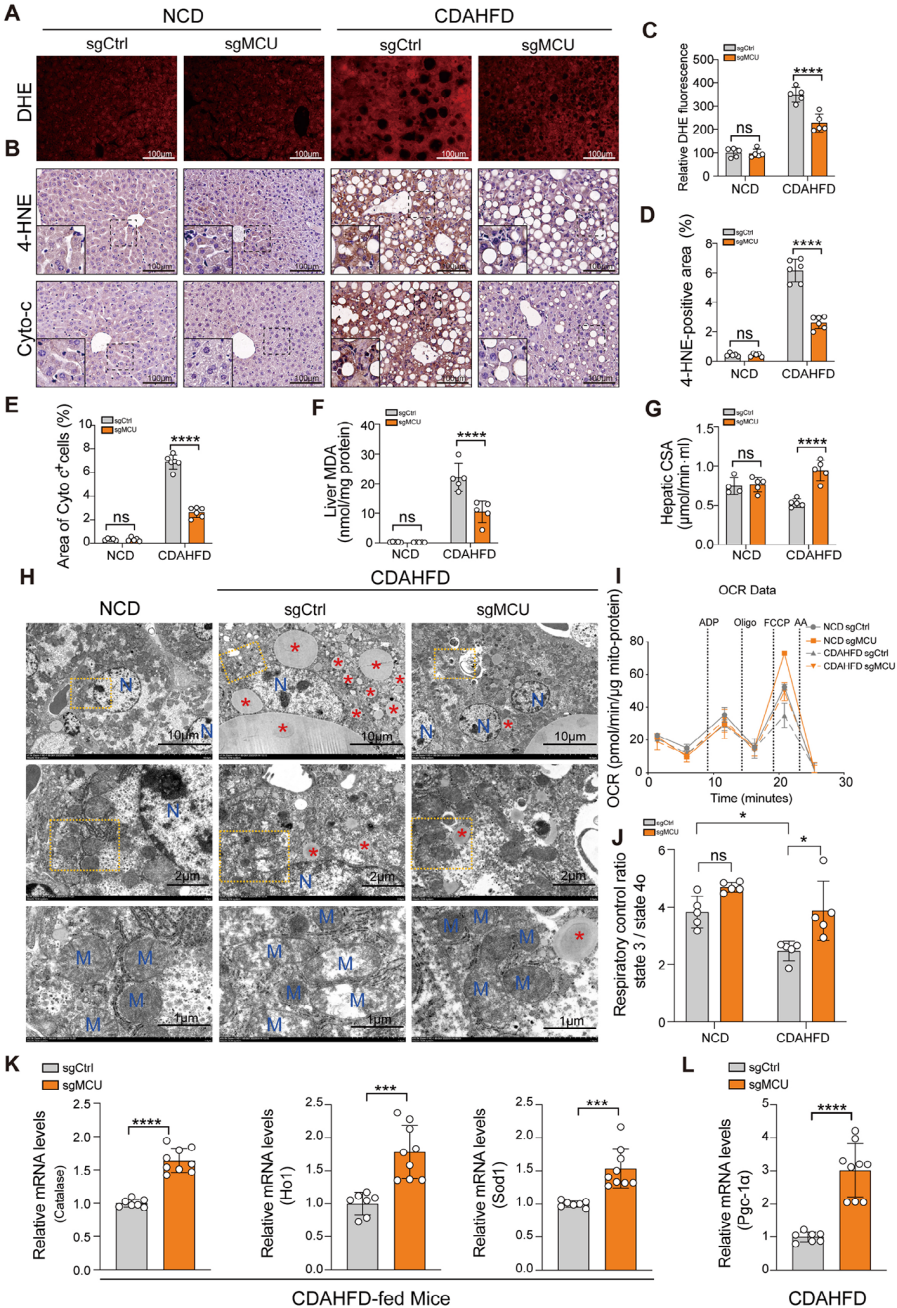
MCU deficiency reduces hepatic oxidative stress and ameliorates mitochondrial dysfunction in experimental MASH. **A** Representative DHE staining in frozen sections of the livers from mice in each group (Scale bars: 100 μm). **B** Immunostaining for 4-HNE and Cytochrome c (Cyto c) of liver sections. Scale bar: 100 μm. Boxed areas in immunostaining images are magnified for the typical morphology. **C** The intensity of red fluorescence of DHE oxidation was quantified (*n* = 5 mice/group). **D** Quantitative analysis of 4-HNE^+^ area per field (%) (*n* = 5–6 mice/group). **E** Quantification of the Cyto c^+^ area (%) in liver sections per field (*n* = 4–5 mice/group). **F** Lipid peroxidation was assessed in terms of MDA formation (*n* = 5 mice/group). **G** Hepatic catalase activity (CSA) was assessed (*n* = 5 mice/group). **H** Representative transmission electron microscopy (TEM) images of the liver sections from indicated mice. Scale bar: 10 μm (upper), 2 μm (middle) and 1 μm (lower). M-mitochondria; ER-endoplasmic reticulum; N-Nucleus; red stars indicate lipid droplets. **I** Liver mitochondria were isolated from indicated mice. Mitochondrial function as measured by oxygen consumption rate (OCR) using the Seahorse analyzer (*n* = 5 mice/group). **J** Respiratory control ratio (RCR) was defined as the state 3/state 4o serving as marker of mitochondrial coupling. **K** Relative mRNA expression of antioxidant marker genes including heme oxygenase 1 (*Ho1*), superoxide dismutase 1 (*Sod1*), *Catalase* in MASH livers was measured by qRT-PCR. The mRNA levels were normalized to β-actin mRNA levels and presented as fold stimulation versus control. **L** Relative mRNA expression of Pgc-1 in MASH livers was measured by qRT-PCR. The mRNA levels were normalized to β-actin and presented as fold stimulation versus control. Data are mean ± SD. Two-way ANOVA with Sidak’s test (D, E) and two-way ANOVA with Tukey’s post-test (C, F, G, J), as well as two-tailed unpaired Student’s *t*-test (K, L) was applied. *P < 0.05, ***P < 0.001, ****P < 0.0001; ns indicates not significant

The production of ROS in MASH livers was assessed using a fluorescent probe DHE bromide. As expected, MASH livers had a substantial increase in fluorescence intensity of DHE and area of DHE^+^ cells, however, these increased ROS were abrogated in MCU-deficient mice (Fig. 8Α and C). We also examined the 4-HNE, a reliable biomarker of lipid peroxidation liver damage [32], by IHC staining and found lowered expression in MCU-deficient MASH livers, accompanied by the reduction of 4-HNE^+^ area (Fig. 8B and D). Indeed, the ameliorating oxidative stress in MCU-deficient MASH mice was further confirmed by the reduced liver MDA (Fig. 8F) and the enhanced hepatic catalase activity (Fig. 8G). Additionally, MCU-deficient mice improved liver antioxidant effect, as confirmed by the elevated mRNA levels of *Catalase*, heme oxygenase 1 (*Ho1*), and superoxide dismutase 1 (*Sod1*) mRNA (Fig. 8K).

Next, we directly assessed the ultrastructural alterations in the MASH livers using TEM. As expected, the characteristic feature of the ultrastructure of the dysmorphic mitochondria was megamitochondria, abnormal shape, and mitochondrial swelling in MASH livers, accompanied by the alterations in hepatocellular ultrastructure, including the gigantic fat droplets, foamy cytoplasmic appearance, and dilated and swollen ER in MASH livers. However, these alterations attenuated in MASH livers from MCU knockdown mice (Fig. 8H). Moreover, we conducted Seahorse experiments to assess OCR and found that mitochondria from MASH mice exhibited a significant reduction in respiratory control ratio (RCR) compared to those from chow-diet mice. However, MCU-deficient mice restored the decreased RCR during MASH (Fig. 8I and J) and the MCU-deficient effects the leakage in the mitochondrial inner membrane to influence RCR (Additional file 1: Fig. S8A and B). IHC staining of Cyto c in liver sections also revealed that the intensity of Cyto c staining was lower in MCU-deficient MASH livers; this result was consistent with the lower of percentage of the Cyto c^+^ cells (Fig. 8B and E). Finally, compared with WT MASH mice, MCU knockout MASH mice exhibited an increase in levels of hepatic *Pgc1α* mRNA (Fig. 8L), indicating targeting MCU enhanced the expression of *Pgc1α* that might induce mitochondrial biogenesis in MASH [13, 38].

Collectively, these results suggest that knockdown of hepatic MCU ameliorates hepatic oxidative stress, ultrastructural alterations, and mitochondrial dysfunction in MASH.

### MCU knockdown inhibits the Hippo/YAP pathway activation and restores AMPK inactivation during MASH development

**Fig. 9:**
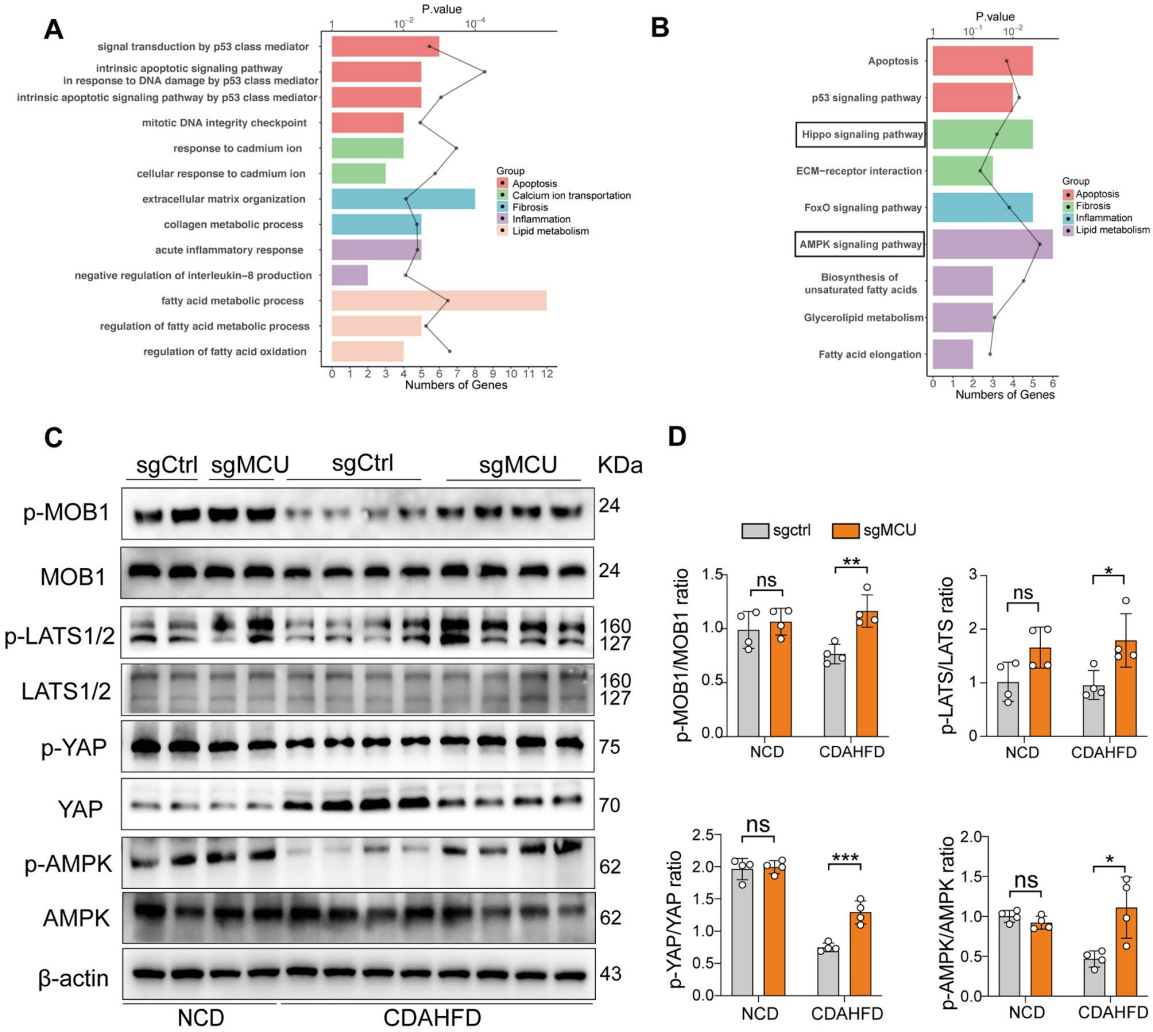
RNA-sequencing analysis and protein immunoblotting indicate the molecular mechanisms involving in targeting MCU for treatment MASH and fibrosis. **A** GO enrichment analysis of the differentially expressed genes (DEGs) in livers between MCU knockdown MASH mice and wild-type (WT) MASH mice. *n* = 3 mice. **B** KEGG pathway and oncogenes enrichment analysis for the DEGs; and top 9 KEGG pathway from enrichment analysis are shown. The false discovery rate (FDR)-adjusted *P-*value was indicated. **C** Western blots for p-MOB1, MOB1, p-LATS1/2, LATS1/2, p-AMPK/AMPK, p-YAP, and YAP in the whole liver from indicated mice; and β-actin serves as a loading control. **D** Densitometric analyses of blots for p-LATS/LATS, p-MOB1/MOB1, p-AMPK/AMPK, and p-YAP/YAP in livers. All data are presented as the mean ± SD. *P*-value are for two-way ANOVA with Tukey’s test. *P < 0.05, **P < 0.01, and ***P < 0.001; ns indicates not significant

To better understand the broader transcriptomic alterations underlying mechanisms by which MCU in MASH and fibrosis, we performed bulk RNA-seq analysis in MASH livers from MCU knockdown mice and WT mice. This analysis displayed that 307 out of the initially screened 26,541 genes were identified as significant DEGs (including 176 upregulated DEGs and 131 downregulated DEGs). Gene Ontology (GO) and gene set enrichment analysis (GSEA) revealed MCU putative target genes involved in the pathogenic processes, including apoptosis, lipid metabolism, inflammation, fibrosis, and Ca^2+^ ion transportation (Fig. 9A and Additional file 1: Fig. S6). Moreover, KEGG pathway analyses identify the top 9 relevant biological pathways among the DEGs affected by hepatic MCU knockdown during MASH pathogenesis, among which two of the most significant pathways were the Hippo pathway and AMPK signaling (Fig. 9B). Altogether, our results indicated that the mechanism of MCU action in MASH and fibrosis might be related to the Hippo pathway and AMPK signaling.

**Fig. 10:**
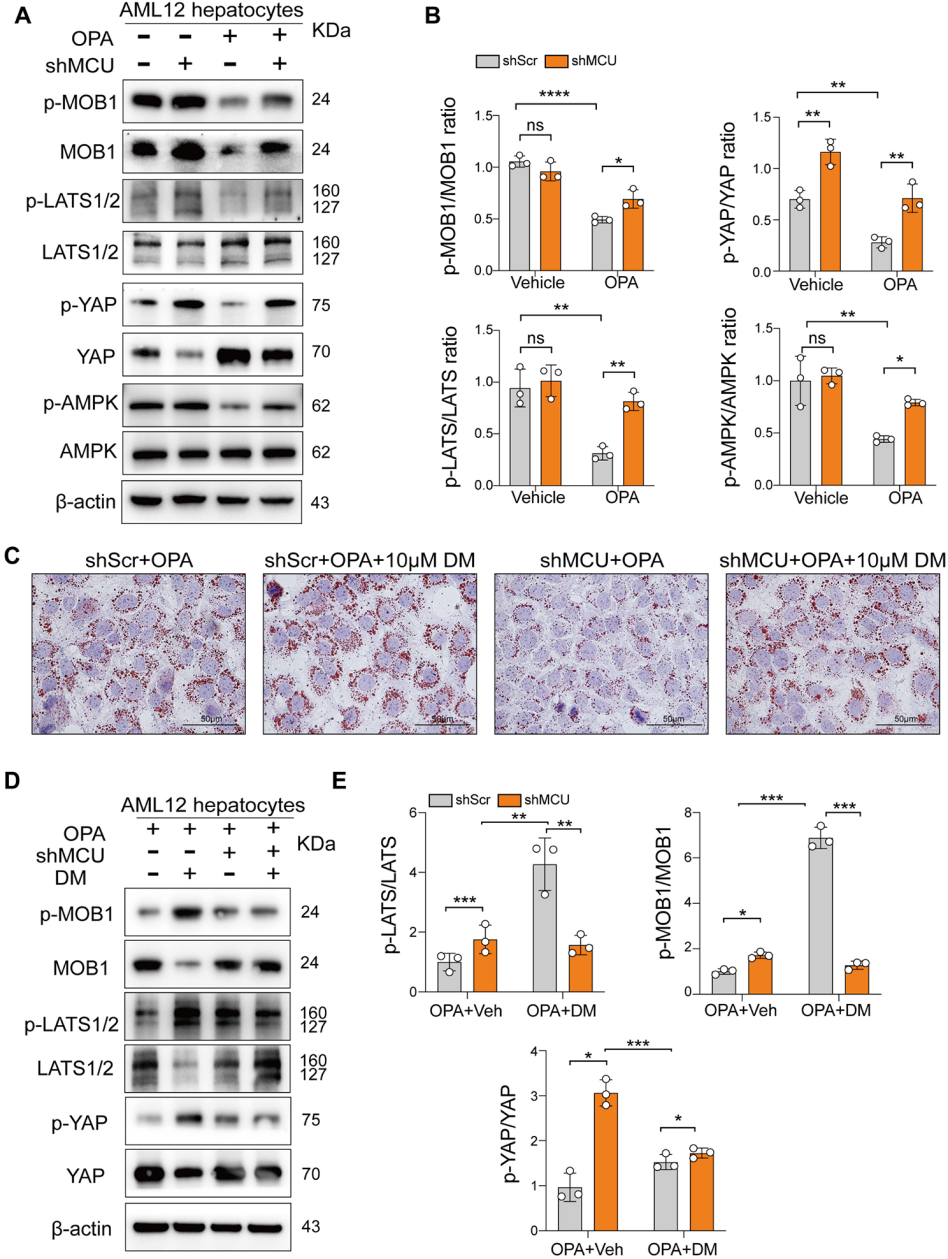
MCU knockdown inhibits the Hippo/YAP signaling and restores AMPK inactivation during MASH development. **A** Western blots for p-MOB1, MOB1, p-LATS1/2, LATS1/2, p-AMPK/AMPK, p-YAP and YAP in MCU silencing AML12 hepatocytes with OPA or vehicle treatment for 24 h; and β-actin as loading control. **B** Densitometric analyses of blots for p-LATS/LATS, p-MOB1/MOB1, p-AMPK/AMPK, and p-YAP/YAP. **C** Representative images of ORO staining in AML12 hepatocytes from indicated treatment. Scale bar: 100 μm. **D** Western blots for p-MOB1, MOB1, p-LATS1/2, LATS1/2, p-YAP and YAP in AML12 hepatocytes; and β-actin as loading control. **E** Densitometric analyses of blots for p-LATS/LATS, p-MOB1/MOB1, and p-YAP/YAP. All data are presented as the mean ± SD. *P*-value are for two-way ANOVA with Tukey’s test. *P < 0.05, **P < 0.01, and ***P < 0.001; ns indicates not significant

To verify the results, the activation state of the large tumor suppressor kinases 1/2 (LATS1/2) and their scaffold protein mob kinase activated 1 (MOB1), as well as YAP in livers was determined by western blot. Compared with chow-diet mice, CDAHFD-feeding mice led to reduced expression of phosphorylated-LATS1/2 (p-LATS1/2) and p-MOB1 in livers, as confirmed by the reduced ratio of p-LATS/LATS and p-MOB1/MOB1 (Fig. 9C and D); however, MCU knockdown MASH mice resorted the reduced levels of p-LATS1/2 and p-MOB1, accompanied by a higher ratio of p-YAP/YAP (Fig. 9C and D). Similarly, cellular model experiments also exhibited that MCU-silenced AML12 hepatocytes abrogated the reduced levels of p-LATS1/2 and p-MOB1 stimulated by OPA, and accompanied by the decreased ratio of p-YAP/YAP (Fig. 10A and B). Further, pharmacological inhibition of the AMPK by Dorsomorphin (DM) could reverse the protective effect of MCU knockdown on lipotoxicity in AML12 hepatocytes (Fig. 10C), which is attributable to the YAP pathway activation by DM (Fig. 10D and E).

Contrary to MCU deficiency, MCU overexpression increased OPA-induced lipid deposition (Additional file 1: Fig. S7A and B), and MCU overexpression in AML12 cells led to a reduced ratio of p-LATS/LAST and accordingly diminishing p-YAP/YAP ratio under OPA treatment (Additional file 1: Fig. S7D and E), indicating overexpressed MCU activated the Hippo/YAP pathway during hepatocellular lipotoxicity.

In addition, consistent with our RNA-seq, AMPK inactivation in MASH livers was confirmed by our western blotting for pAMPK and AMPK, but this inactivation was significantly blocked in MCU-deficient MASH mice (Fig. 9C and D). Accordingly, OPA treatment AML12 hepatocytes led to inactivation of AMPK as assessed by western blotting for pAMPK and AMPK, however, MCU-deficient AML12 hepatocytes stimulated by OPA restored the activated AMPK (Fig. 10A and B). But MCU overexpression in AML12 hepatocytes led to inactivation of AMPK (Additional file 1: Fig. S7D and E). Given the AMPK activation plays a critical role in the coordination of cell metabolism and function that can directive phosphorylate YAP [39, 40], our data indicate that blockage of MCU led to YAP phosphorylation, at least in part, due to AMPK-dependent LATS1/2 activation.

Taken together, our results strongly suggest that knockdown MCU inhibits the Hippo/YAP pathway activation and restores AMPK inactivation during MASH pathogenesis.

## Discussion

In this study, we revealed that hepatic MCU plays a vital role in the pathology of MASH and fibrosis. Using a CDAHFD-fed mouse model to rapidly develop MASH with progressive liver fibrosis [29, 30], we have shown that hepatic MCU knockdown robustly mitigated multiple aspects of the disease, including steatosis, inflammation, and fibrosis, as well as improved hepatic oxidative stress and mitochondrial dysfunction.

Recent studies have demonstrated that MCU regulates the entry of Ca^2+^ into mitochondrial matrix that plays a key role in Ca^2+^ homeostasis, however, the precise role and potential mechanism of MCU in steatohepatitis and its related liver fibrosis waited to be elucidated. Here, we affirm that MCU expression is minimal in healthy liver samples of humans and mice. But MCU expression is strikingly enhanced in patients with simple steatosis and steatohepatitis (Fig. 1) as well as in both livers and primary hepatocytes of murine models of MASH (Fig. 2). Supported our results, previous studies have displayed that MCU expression was higher in the *ob*/*ob* mice [24], HCC human tissue and hepatocyte cell lines [25, 41, 42]. Notably, MCU is predominantly distributed in hepatocytes of MASH livers from human samples (Fig. 1B) and mice (Fig. 2A, C). Moreover, in our cellular models’ study, we further demonstrate that lipotoxic stress remarkably induced MCU expression (Additional file 1: Fig. S2G, H). The expression of MCU is controlled by the Ca^2+^-regulating transcription factor cyclic AMP response element-binding protein, which binds directly to the promoter of MCU to stimulate its transcription. Therefore, our data indicates that MCU plays contributes to the development or progression of a wide variety of diseases including metabolism disorders by altering or controlling its expression and activity [11, 43].

Here, our in vitro experiments reveal that MCU silencing in AML12 hepatocytes abrogated mitochondrial Ca^2+^ uptake (Fig. 3A and B), subsequently decreasing lipid accumulation (Fig. 6A and B), thereby restraining OPA-induced hepatocyte apoptotic death (Fig. 7A-C). Furthermore, we demonstrate that the knockdown of MCU robustly attenuated reduced lipid droplets in hepatocytes in MASH mice (Fig. 4). Thus, our findings indicate the aberrant elevated MCU expression plays a pathological role in hepatosteatosis during MASH development both in vivo and in vitro. In line with our results, blockade of MCU by Ru360 administration or genetic ablation diminished hepatic lipid accumulation in high-fat diet-fed mice [44]. MCU ablation or inhibition attenuated cadmium-induced excessive mitophagy, thereby rescuing mitochondrial dysfunction and enhancing hepatocyte viability [45]. A recent investigation by Hu et al. [46] showed that MCU-mediated uptake of ferrous iron (Fe^2+^) into mitochondria during acetaminophen-induced hepatotoxicity triggered mitochondrial depolarization and cell death. Notably, a more recent study by LaMoia et al. [47] revealed that liver-specific deletion of MCU increased mitochondrial oxidation and reduced hepatic lipid accumulation. Surprisingly, in contrast to the findings by Tomar et al. [48] deletion of MCU gene in mouse liver inhibited MCU-mediated Ca^2+^ uptake but aggravated hepatic lipid deposition. However, for reasons not well understood, the discrepancies observed in these studies have been attributed to differences in phenotypes of MCU knockout animal models, dietary intake, partial or complete loss of MCU, and genetic background of the animals [18, 43, 46, 47, 49].

Previous studies have shown that MCU-mediated Ca^2+^ overload leads to mitochondrial dysfunction under oxidative stress [26], which plays an important role in the pathogenesis of MASH [5–7, 32]. Remarkably, we find that partial loss of hepatic MCU was associated with attenuating hepatic oxidative damage and mitochondrial dysfunction in MASH livers and in hepatocytes challenged by lipotoxicity. Supported by this result, the knockdown of MCU was confirmed by the improved ultrastructural alterations (Fig. 8H) and increased mitochondrial biogenesis (Fig. 8L). Notably, in keeping with studies exhibiting that the levels of *Pgc1α* mRNA reduced during MASH development [38], we found that the decreased levels of *Pgc1α* mRNA in MASH livers were abrogated by hepatic MCU knockdown mice (Fig. 8L). Similarly, mRNA levels of *Pgc1α* were lowered in our cellular models of MASLD, while silencing MCU restored the reduced *Pgc1α* mRNA in hepatocytes exposed to OPA toxicity (Fig. S7C). Consistent with previous evidence that MCU silencing or deletion prevents mitochondrial Ca^2+^ overload and apoptotic cell death [17, 23, 26], MCU knockdown in hepatocytes indeed led to a marked reduction in the markers of apoptosis (Fig. 7A-C, E, F).

Furthermore, downregulation of MCU inhibited macrophage infiltration into the livers and limited hepatic inflammatory response in mice during MASH development (Fig. 4C and F). In vitro analysis, silencing of MCU in AML12 cells led to reducing the levels of *Il1β* and *Il6* mRNA expression during lipotoxicity (Additional file 1: Fig. S3). Notably, previous studies revealed that higher MCU activity triggers NLRP3 inflammasome activation contributing to inflammation and cystic fibrosis [50]. Knockout of MCU suppressed mitochondrial Ca^2+^ uptake and subsequently led to a reduced expression of *Tnfα* and *Il6* mRNA in HepG2 cells stimulated by high glucose [51]. Macrophage inflammatory activation and cytokine release under lipotoxicity triggered HSC activation and promoted liver fibrosis [1, 4]. In accordance with limiting liver inflammatory response, knockdown of hepatic MCU limited liver fibrosis in MASH. Similarly, accumulating evidence has exhibited that MCU dysfunction and subsequent mitochondrial Ca^2+^ overload play a major contribution in multiple organ fibrosis [15, 21, 22]. However, loss of MCU in alveolar type 2 cells caused immune lung injury and promoted mortality [21]. Interestingly, fibroblast-specific deletion of MCU in adult mice induced myofibroblast formation, therefore leading to enhanced cardiac fibrosis [20]. Thereby, these data have indicated that the precise roles of MCU are complex and differ depending on the cell types and distinct organs during injury and fibrogenesis.

Mechanistically, we described a novel molecular function of MCU linking to the Hippo/YAP pathway and AMPK signaling (Figs. 9 and 10). It has been established that the Hippo/YAP pathway has been implicated in the pathology of MASH and liver fibrosis [39, 52]. Moreover, a previous report that indicated the crosstalk between MCU and the Hippo signaling in mammalian cell lines [53]. Notably, impaired AMPK activity has been shown to promote MASH and fibrosis [54–56]. Here, we indeed find that mice fed CDAHFD resulted in AMPK inactivation as confirmed by our RNA-seq and western blotting analysis (Fig. 9B-D). Our data also exhibited that inhibition of AMPK led to loss of protective effect on hepatosteatosis of MCU knockdown (Fig. 10C). Moreover, AMPK-mediated phosphorylation of Ser57 on MCU could positively regulate mitochondrial Ca^2+^ entry, enhancing mitochondrial respiration and energy production [57]. Interestingly, a previous study exhibited cellular energy stress-induced YAP phosphorylation, which is partially due to AMPK-dependent LATS activation [40, 58]. Thus, our findings both in vivo and in vitro indicate that knockdown of MCU might restore AMPK activation and promote the Hippo/YAP pathway inactivation in MASH development.

This work presented here has several limitations and open questions remain. First, only one MASH model was used to decipher the effect of knockdown hepatic MCU on liver fibrosis; and whether knockdown of hepatic MCU could promote fibrosis resolution is a question to be elucidated in further work. Second, the MCU complex serves as an adaptive mechanism [57], we only knockdown the essential component of the MCU complex in mice to explore the MCU’s role in MASH development, and several regulators of the MCU complex such as EMRE, MICU1, MICU2, and MCUb might be also involved in this pathogenesis that did not evaluate in this study. Third, our data only addicted the Hippo/YAP pathway and AMPK activity related to MCU-mediated Ca^2+^ signaling. However, whether and how MCU directly or indirectly regulates the Hippo/YAP pathway needs to be further clarified. Thus, future research is required to better understand how MCU-mediated mitochondrial Ca^2+^ signaling is regulated during pathological conditions and how this impacts liver metabolism, which is crucial to the discovery of novel therapeutic targets for MASH.

## Conclusions

Our findings demonstrate that MCU was up-regulated in the liver from humans and mice with MASH; and knockdown of hepatic MCU attenuates MASH and fibrosis via regulating AMPK activation and the Hippo/YAP pathway. Thus, MCU may represent a potential therapeutic target to mitigate MASH and fibrosis.

## Electronic supplementary material

Below is the link to the electronic supplementary material.

Supplementary Material 1

Supplementary Material 2

## Data Availability

The datasets generated during and/or analyzed during the current study are available from the corresponding author upon reasonable request.

## Abbreviations

AAV: Adeno-associated virus
ACC: Acetyl-CoA carboxylase
AMPK: AMP-activated protein kinase
αSMA: Alpha-smooth actin
BSA: Bovine serum albumin
Ca^2+^: Calcium
CDAHFD: Choline-deficient, L-amino acid-defined high-fat diet
CREB: cAMP response element binding protein
DEGs: Differentially expressed genes
FAS: Fatty acid synthase
FBS: Fetal bovine serum
GO: Gene Ontology
GSEA: Gene set enrichment analysis
HFCD: High-fat/calorie diet
HSCs: Hepatic stellate cells
HCC: Hepatocellular carcinoma
HNF4: Hepatic nuclear factor 4
Ho1: Heme oxygenase 1
ER: Endoplasmic reticulum
KEGG: Kyoto Encyclopedia of Genes and Genomes
LATS1/2: Large tumour suppressor kinases 1/2
MASH: Metabolic-dysfunction associated steatohepatitis
MASLD: Metabolic dysfunction-associated steatotic liver disease
MCU: Mitochondrial Ca^2+^ uniporter
MMP: Mitochondrial membrane potential
MOB1: Mob kinase activated 1
mtROS: Mitochondrial ROS
NASH: Nonalcoholic steatohepatitis
NAFLD: Nonalcoholic fatty liver disease
OPA: Oleic and palmitic acid
PPARγ: Peroxisome proliferator-activated receptor gamma
SREBF1: Sterol regulatory element binding transcription factor 1
RCR: Respiratory control ratio
SOD1: Superoxide dismutase 1
TNFα: Tumor necrosis factor alpha
CPT1A: Recombinant Carnitine Palmitoyltransferase 1 A
COX IV: Cytochrome c oxidase subunit IV

## References

[1] Wang S, Friedman SL. Found in translation-fibrosis in metabolic dysfunction-associated steatohepatitis (MASH). Sci Transl Med. 2023;15:eadi0759. 37792957 10.1126/scitranslmed.adi0759 PMC10671253

[2] Rinella ME, Lazarus JV, Ratziu V, Francque SM, Sanyal AJ, Kanwal F, et al. A multisociety Delphi consensus statement on new fatty liver disease nomenclature. Hepatology. 2023;78:1966–86. 37363821 10.1097/HEP.0000000000000520 PMC10653297

[3] Parola M, Pinzani M. Liver fibrosis in NAFLD/NASH: from pathophysiology towards diagnostic and therapeutic strategies. Mol Aspects Med. 2024;95:101231. 38056058 10.1016/j.mam.2023.101231

[4] Schuster S, Cabrera D, Arrese M, Feldstein AE. Triggering and resolution of inflammation in NASH. Nat Rev Gastroenterol Hepatol. 2018;15:349–64. 29740166 10.1038/s41575-018-0009-6

[5] Buzzetti E, Pinzani M, Tsochatzis EA. The multiple-hit pathogenesis of non-alcoholic fatty liver disease (NAFLD). Metabolism. 2016;65:1038–48. 26823198 10.1016/j.metabol.2015.12.012

[6] Kim JY, Garcia-Carbonell R, Yamachika S, Zhao P, Dhar D, Loomba R, et al. ER stress drives Lipogenesis and Steatohepatitis via Caspase-2 activation of S1P. Cell. 2018;175:133–45. e115. 30220454 10.1016/j.cell.2018.08.020 PMC6159928

[7] Sharma S, Le Guillou D, Chen JY. Cellular stress in the pathogenesis of nonalcoholic steatohepatitis and liver fibrosis. Nat Rev Gastroenterol Hepatol. 2023;20:662–78. 37679454 10.1038/s41575-023-00832-w

[8] Humbert A, Lefebvre R, Nawrot M, Caussy C, Rieusset J. Calcium signalling in hepatic metabolism: Health and diseases. Cell Calcium. 2023;114:102780. 37506596 10.1016/j.ceca.2023.102780

[9] Brumer RP, Correa-Velloso JC, Thomas SJ, Sandiford OA, Thomas AP, Bartlett PJ. Short-term high-fat diet feeding of mice suppresses catecholamine-stimulated ca (2+) signalling in hepatocytes and intact liver. J Physiol. 2023;601:1383–405. 36864773 10.1113/JP283691

[10] Marchi S, Patergnani S, Missiroli S, Morciano G, Rimessi A, Wieckowski MR, et al. Mitochondrial and endoplasmic reticulum calcium homeostasis and cell death. Cell Calcium. 2018;69:62–72. 28515000 10.1016/j.ceca.2017.05.003

[11] Garbincius JF, Elrod JW. Mitochondrial calcium exchange in physiology and disease. Physiol Rev. 2022;102:893–992. 34698550 10.1152/physrev.00041.2020 PMC8816638

[12] Alevriadou BR, Patel A, Noble M, Ghosh S, Gohil VM, Stathopulos PB, Madesh M. Molecular nature and physiological role of the mitochondrial calcium uniporter channel. Am J Physiol Cell Physiol. 2021;320:C465–82. 33296287 10.1152/ajpcell.00502.2020 PMC8260355

[13] Baughman JM, Perocchi F, Girgis HS, Plovanich M, Belcher-Timme CA, Sancak Y, et al. Integrative genomics identifies MCU as an essential component of the mitochondrial calcium uniporter. Nature. 2011;476:341–5. 21685886 10.1038/nature10234 PMC3486726

[14] Mallilankaraman K, Doonan P, Cardenas C, Chandramoorthy HC, Muller M, Miller R, et al. MICU1 is an essential gatekeeper for MCU-mediated mitochondrial ca(2+) uptake that regulates cell survival. Cell. 2012;151:630–44. 23101630 10.1016/j.cell.2012.10.011 PMC3486697

[15] Lozano O, Marcos P, Salazar-Ramirez FJ, Lazaro-Alfaro AF, Sobrevia L, Garcia-Rivas G. Targeting the mitochondrial ca(2+) uniporter complex in cardiovascular disease. Acta Physiol (Oxf). 2023;237:e13946. 36751976 10.1111/apha.13946

[16] Watanabe A, Maeda K, Nara A, Hashida M, Ozono M, Nakao A, et al. Quantitative analysis of mitochondrial calcium uniporter (MCU) and essential MCU regulator (EMRE) in mitochondria from mouse tissues and HeLa cells. FEBS Open Bio. 2022;12:811–26. 35060355 10.1002/2211-5463.13371 PMC8972046

[17] Woods JJ, Wilson JJ. Inhibitors of the mitochondrial calcium uniporter for the treatment of disease. Curr Opin Chem Biol. 2020;55:9–18. 31869674 10.1016/j.cbpa.2019.11.006

[18] Nemani N, Shanmughapriya S, Madesh M. Molecular regulation of MCU: implications in physiology and disease. Cell Calcium. 2018;74:86–93. 29980025 10.1016/j.ceca.2018.06.006 PMC6119482

[19] Li S, Chen J, Liu M, Chen Y, Wu Y, Li Q, et al. Protective effect of HINT2 on mitochondrial function via repressing MCU complex activation attenuates cardiac microvascular ischemia-reperfusion injury. Basic Res Cardiol. 2021;116:65. 34914018 10.1007/s00395-021-00905-4 PMC8677646

[20] Lombardi AA, Gibb AA, Arif E, Kolmetzky DW, Tomar D, Luongo TS, et al. Mitochondrial calcium exchange links metabolism with the epigenome to control cellular differentiation. Nat Commun. 2019;10:4509. 31586055 10.1038/s41467-019-12103-x PMC6778142

[21] Islam MN, Gusarova GA, Das SR, Li L, Monma E, Anjaneyulu M, et al. The mitochondrial calcium uniporter of pulmonary type 2 cells determines severity of acute lung injury. Nat Commun. 2022;13:5837. 36192486 10.1038/s41467-022-33543-y PMC9529882

[22] Sebag SC, Koval OM, Paschke JD, Winters CJ, Comellas AP, Grumbach IM. Inhibition of the mitochondrial calcium uniporter prevents IL-13 and allergen-mediated airway epithelial apoptosis and loss of barrier function. Exp Cell Res. 2018;362:400–11. 29225050 10.1016/j.yexcr.2017.12.003 PMC6330015

[23] De Mario A, Tosatto A, Hill JM, Kriston-Vizi J, Ketteler R, Vecellio Reane D, et al. Identification and functional validation of FDA-approved positive and negative modulators of the mitochondrial calcium uniporter. Cell Rep. 2021;35:109275. 34161774 10.1016/j.celrep.2021.109275 PMC8242467

[24] Arruda AP, Pers BM, Parlakgul G, Guney E, Inouye K, Hotamisligil GS. Chronic enrichment of hepatic endoplasmic reticulum-mitochondria contact leads to mitochondrial dysfunction in obesity. Nat Med. 2014;20:1427–35. 25419710 10.1038/nm.3735 PMC4412031

[25] Jin C, Kumar P, Gracia-Sancho J, Dufour JF. Calcium transfer between endoplasmic reticulum and mitochondria in liver diseases. FEBS Lett. 2021;595:1411–21. 33752262 10.1002/1873-3468.14078

[26] Ali ES, Rychkov GY, Barritt GJ. Deranged hepatocyte intracellular ca(2+) homeostasis and the progression of non-alcoholic fatty liver disease to hepatocellular carcinoma. Cell Calcium. 2019;82:102057. 31401389 10.1016/j.ceca.2019.102057

[27] Bhowmick S, Singh V, Jash S, Lal M, Sinha Roy S. Mitochondrial metabolism and calcium homeostasis in the development of NAFLD leading to hepatocellular carcinoma. Mitochondrion. 2021;58:24–37. 33581332 10.1016/j.mito.2021.01.007

[28] Rieusset J. Endoplasmic reticulum-mitochondria calcium signaling in hepatic metabolic diseases. Biochim Biophys Acta Mol Cell Res. 2017;1864:865–76. 28064001 10.1016/j.bbamcr.2017.01.001

[29] Matsumoto M, Hada N, Sakamaki Y, Uno A, Shiga T, Tanaka C, et al. An improved mouse model that rapidly develops fibrosis in non-alcoholic steatohepatitis. Int J Exp Pathol. 2013;94:93–103. 23305254 10.1111/iep.12008 PMC3607137

[30] Yao Q, Li S, Li X, Wang F, Tu C. Myricetin modulates macrophage polarization and mitigates liver inflammation and fibrosis in a murine model of nonalcoholic steatohepatitis. Front Med (Lausanne). 2020;7:71. 32195263 10.3389/fmed.2020.00071 PMC7065264

[31] Liu XJ, Duan NN, Liu C, Niu C, Liu XP, Wu J. Characterization of a murine nonalcoholic steatohepatitis model induced by high fat high calorie diet plus fructose and glucose in drinking water. Lab Invest. 2018;98:1184–99. 29959418 10.1038/s41374-018-0074-z

[32] Li S, Li X, Chen F, Liu M, Ning L, Yan Y, et al. Nobiletin mitigates hepatocytes death, liver inflammation, and fibrosis in a murine model of NASH through modulating hepatic oxidative stress and mitochondrial dysfunction. J Nutr Biochem. 2022;100:108888. 34695558 10.1016/j.jnutbio.2021.108888

[33] Lan T, Jiang S, Zhang J, Weng Q, Yu Y, Li H, et al. Breviscapine alleviates NASH by inhibiting TGF-beta-activated kinase 1-dependent signaling. Hepatology. 2022;76:155–71. 34717002 10.1002/hep.32221 PMC9299589

[34] Bi G, Bian Y, Liang J, Yin J, Li R, Zhao M, et al. Pan-cancer characterization of metabolism-related biomarkers identifies potential therapeutic targets. J Transl Med. 2021;19:219. 34030708 10.1186/s12967-021-02889-0 PMC8142489

[35] Ritchie ME, Phipson B, Wu D, Hu Y, Law CW, Shi W, et al. Limma powers differential expression analyses for RNA-sequencing and microarray studies. Nucleic Acids Res. 2015;43:e47. 25605792 10.1093/nar/gkv007 PMC4402510

[36] Subramanian A, Tamayo P, Mootha VK, Mukherjee S, Ebert BL, Gillette MA, et al. Gene set enrichment analysis: a knowledge-based approach for interpreting genome-wide expression profiles. Proc Natl Acad Sci U S A. 2005;102:15545–50. 16199517 10.1073/pnas.0506580102 PMC1239896

[37] Rogers GW, Brand MD, Petrosyan S, Ashok D, Elorza AA, Ferrick DA, et al. High throughput microplate respiratory measurements using minimal quantities of isolated mitochondria. PLoS ONE. 2011;6:e21746. 21799747 10.1371/journal.pone.0021746 PMC3143121

[38] Mansouri A, Gattolliat CH, Asselah T. Mitochondrial dysfunction and signaling in Chronic Liver diseases. Gastroenterology. 2018;155:629–47. 30012333 10.1053/j.gastro.2018.06.083

[39] Salloum S, Jeyarajan AJ, Kruger AJ, Holmes JA, Shao T, Sojoodi M, et al. Fatty acids activate the Transcriptional Coactivator YAP1 to promote liver fibrosis via p38 mitogen-activated protein kinase. Cell Mol Gastroenterol Hepatol. 2021;12:1297–310. 34118488 10.1016/j.jcmgh.2021.06.003 PMC8463869

[40] Mo JS, Meng Z, Kim YC, Park HW, Hansen CG, Kim S, et al. Cellular energy stress induces AMPK-mediated regulation of YAP and the Hippo pathway. Nat Cell Biol. 2015;17:500–10. 25751140 10.1038/ncb3111 PMC4380774

[41] Li CJ, Lin HY, Ko CJ, Lai JC, Chu PY. A novel biomarker driving poor-prognosis Liver Cancer: overexpression of the mitochondrial calcium gatekeepers. Biomedicines 2020;8. 10.3390/biomedicines8110451 PMC7693594 33114428

[42] Ren S, Wang J, Dong Z, Li J, Ma Y, Yang Y, et al. Perfluorooctane sulfonate induces ferroptosis-dependent non-alcoholic steatohepatitis via autophagy-MCU-caused mitochondrial calcium overload and MCU-ACSL4 interaction. Ecotoxicol Environ Saf. 2024;280:116553. 38850699 10.1016/j.ecoenv.2024.116553

[43] D’Angelo D, Rizzuto R. The mitochondrial calcium Uniporter (MCU): Molecular Identity and Role in Human diseases. Biomolecules. 2023;13(9):1304. 37759703 10.3390/biom13091304 PMC10526485

[44] Zhang Z, Luo Z, Yu L, Xiao Y, Liu S. Ruthenium 360 and mitoxantrone inhibit mitochondrial calcium uniporter channel to prevent liver steatosis induced by high-fat diet. Br J Pharmacol. 2022;179:2678–96. 34862596 10.1111/bph.15767

[45] Liu C, Li HJ, Duan WX, Duan Y, Yu Q, Zhang T, et al. MCU Upregulation overactivates Mitophagy by promoting VDAC1 dimerization and ubiquitination in the hepatotoxicity of Cadmium. Adv Sci (Weinh). 2023;10:e2203869. 36642847 10.1002/advs.202203869 PMC9982555

[46] Hu J, Nieminen AL, Weemhoff JL, Jaeschke H, Murphy LG, Dent JA, et al. The mitochondrial calcium uniporter mediates mitochondrial Fe^2+^ uptake and hepatotoxicity after acetaminophen. Toxicol Appl Pharmacol. 2023;479:116722. 37848124 10.1016/j.taap.2023.116722 PMC10872750

[47] LaMoia TE, Hubbard BT, Guerra MT, Nasiri A, Sakuma I, Kahn M et al. Cytosolic calcium regulates hepatic mitochondrial oxidation, intrahepatic lipolysis, and gluconeogenesis via CAMKII activation. Cell Metab. 2024 Aug 13:S1550-4131(24)00287-0. doi: 10.1016/j.cmet.2024.07.016. Epub ahead of print. PMID: 39153480. 10.1016/j.cmet.2024.07.016 PMC11446666 39153480

[48] Tomar D, Jana F, Dong Z, Quinn WJ 3rd, Jadiya P, Breves SL, et al. Blockade of MCU-Mediated ca(2+) Uptake perturbs lipid metabolism via PP4-Dependent AMPK Dephosphorylation. Cell Rep. 2019;26:3709–e37253707. 30917323 10.1016/j.celrep.2019.02.107 PMC6512325

[49] Arduino DM, Perocchi F. Pharmacological modulation of mitochondrial calcium homeostasis. J Physiol. 2018;596:2717–33. 29319185 10.1113/JP274959 PMC6046065

[50] Rimessi A, Bezzerri V, Patergnani S, Marchi S, Cabrini G, Pinton P. Mitochondrial Ca2+-dependent NLRP3 activation exacerbates the Pseudomonas aeruginosa-driven inflammatory response in cystic fibrosis. Nat Commun. 2015;6:6201. 25648527 10.1038/ncomms7201

[51] Panahi G, Pasalar P, Zare M, Rizzuto R, Meshkani R. MCU-knockdown attenuates high glucose-induced inflammation through regulating MAPKs/NF-kappaB pathways and ROS production in HepG2 cells. PLoS ONE. 2018;13:e0196580. 29709004 10.1371/journal.pone.0196580 PMC5927441

[52] Alsamman S, Christenson SA, Yu A, Ayad NME, Mooring MS, Segal JM, et al. Targeting acid ceramidase inhibits YAP/TAZ signaling to reduce fibrosis in mice. Sci Transl Med. 2020;12(557):eaay8798. 32817366 10.1126/scitranslmed.aay8798 PMC7976849

[53] Meng K, Hu Y, Wang D, Li Y, Shi F, Lu J, et al. EFHD1, a novel mitochondrial regulator of tumor metastasis in clear cell renal cell carcinoma. Cancer Sci. 2023;114:2029–40. 36747492 10.1111/cas.15749 PMC10154798

[54] Zhao P, Sun X, Chaggan C, Liao Z, In Wong K, He F, et al. An AMPK-caspase-6 axis controls liver damage in nonalcoholic steatohepatitis. Science. 2020;367:652–60. 32029622 10.1126/science.aay0542 PMC8012106

[55] Zhang J, Muise ES, Han S, Kutchukian PS, Costet P, Zhu Y, et al. Molecular profiling reveals a common metabolic signature of tissue fibrosis. Cell Rep Med. 2020;1(4):100056. 33205063 10.1016/j.xcrm.2020.100056 PMC7659620

[56] Li X, Wang H. Multiple organs involved in the pathogenesis of non-alcoholic fatty liver disease. Cell Biosci. 2020;10(1):140. 33372630 10.1186/s13578-020-00507-y PMC7720519

[57] Vecellio Reane D, Serna JDC, Raffaello A. Unravelling the complexity of the mitochondrial Ca2 + uniporter: regulation, tissue specificity, and physiological implications. Cell Calcium. 2024;121:102907. 38788256 10.1016/j.ceca.2024.102907

[58] Koo JH, Guan KL. Interplay between YAP/TAZ and metabolism. Cell Metab. 2018;28:196–206. 30089241 10.1016/j.cmet.2018.07.010

