# Supplementary material for: Knockdown of hepatic mitochondrial calcium uniporter mitigates MASH and fibrosis in mice
Source: Cell Biosci. 2024 Nov 10;14:135. doi: 10.1186/s13578-024-01315-4 (PMC11550531; doi:10.1186/s13578-024-01315-4)
Supplement: Supplementary file 1 — Supplementary Material 1 [file 13578_2024_1315_MOESM1_ESM.docx]

**Supplementary Data**

**Knockdown of hepatic mitochondrial calcium uniporter mitigates MASH and fibrosis in mice**

Shuyu Li^1,†^, Fangyuan Chen^1,†^, Min Liu^2^, Yajun Zhang^2^, Jingjing Xu^3^, Xi Li^4^, Zhiyin Shang^2^, Shaoping Huang^2^, Shu Song^3,*^, Chuantao Tu^2,*^

^1^Department of Gastroenterology and Hepatology, Zhongshan Hospital, Fudan University, Shanghai, 200032, China

^2^Department of Gastroenterology, Shanghai Public Health Clinical Center, Fudan University, Shanghai, 201508, China.

^3^Department of Pathology, Shanghai Public Health Clinical Center, Fudan University, Shanghai, 201508, China.

^4^Department of Geriatrics, Zhongshan Hospital, Fudan University, Shanghai, 200032, China.

^†^Shuyu Li and Fangyuan Chen contributed equally to this work.

**Correspondence**

*Correspondence author.

1. mail address: [songshu@shaphc.org](mailto:songshu@shaphc.org) (S. Song); [tuchuantao@shaphc.org](mailto:tuchuantao@shaphc.org) (C. Tu)

**This file includes:**

Tables S1 to S4

Figures S1 to S8

**Table**

**Table S1** All antibodies in this study

| Antibody | Catalog Number | Vendors |
| --- | --- | --- |
| anti-α-SMA | #19245 | Cell Signaling Technology |
| anti-MCU | #26312-1-AP | Proteintech Group |
| anti-4-hydroxynonenal | #ab48506 | Abcam |
| anti-Cyto C | #ab133504 | Abcam |
| anti-F4/80 | #70076 | Cell Signaling Technology |
| anti-cleaved Caspase-3 | #9664 | Cell Signaling Technology |
| anti-HNF4 | #3113 | Cell Signaling Technology |
| anti-caspase3 | #9662 | Cell Signaling Technology |
| anti-β-Actin | #T40104 | Abmart |
| anti-p-AMPK | #2531 | Cell Signaling Technology |
| anti-AMPK | #2532 | Cell Signaling Technology |
| anti-Caspase3 | #19677-1-AP | Proteintech Group |
| anti-COX IV | # T40110 | Abmart |
|  |  |  |

**Table S2** The oligo sequences for siRNA used in this study

|  | Sequence (5’~3’) |
| --- | --- |
| siMCU1 (646) | GGAGAAGGUACGAAUUGAATT |
|  | UUCAAUUCGUACCUUCUCCTT |
| siMCU2 (850) | GCGCCAGGAAUAUGUUUAUTT |
|  | AUAAACAUAUUCCUGGCGCTT |
| siMCU3 (971) | CAGGCAGAAAUGGAUCUUATT |
|  | UAAGAUCCAUUUCUGCCUGTT |
| Scramble | UUCUCCGAACGUGUCACGUTT |
|  | ACGUGACACGUUCGGAGAATT |

**Table S3** Clinical and laboratory characteristics of patients and health control

|  | HC(n=11) | | | NASLD(n=13) | | NASH (n=13) | | |
| --- | --- | --- | --- | --- | --- | --- | --- | --- |
|  | mean | SD | mean | | SD | | mean | SD |
| M/F | 0.6364 | 0.5045 | 0.5385 | | 0.5189 | | 0.5385 | 0.5189 |
| Age(y) | 54.73 | 13.94 | 52.31 | | 12.72 | 59.15 | | 12.23 |
| Height(cm) | 1.661 | 0.06441 | 1.695 | | 0.08363 | | 1.675 | 0.1057 |
| Weight(kg) | 63.95 | 12.88 | 78.19 | | 17.78 | | 80.13 | 23.27 |
| BMI | 23.02 | 3.357 | 27.59 | | 4.531 | | 28.13 | 4.686 |
| Liver Stiffness(kPa) | 4.791 | 1.906 | 7.323 | | 2.038 | | 10.53 | 5.913 |
| PLT(*10^9/L) | 188.5 | 42.37 | 204.2 | | 66.32 | | 231.3 | 52.1 |
| INR | 0.99 | 0.0506 | 0.9938 | | 0.05026 | | 1.015 | 0.1468 |
| PT (s) | 11.67 | 0.5039 | 13.13 | | 0.5218 | | 12.18 | 1.598 |
| ALT (U/L) | 14.55 | 5.973 | 73.77 | | 52.51 | | 35.69 | 37.74 |
| AST (U/L) | 14.45 | 2.841 | 45.85 | | 27.97 | | 24.46 | 14.68 |
| ALP (U/L) | 65.18 | 14.39 | 88.31 | | 22.69 | | 70.23 | 3.217 |
| γGT (U/L) | 23.82 | 13.83 | 104.9 | | 114.5 | | 45.23 | 47.04 |
| TBil(μmol/L) | 11.87 | 4.723 | 18.77 | | 11.95 | | 12.58 | 9.177 |
| ALB(g/L) | 38.06 | 12.16 | 43.16 | | 10.73 | | 43.75 | 4.479 |
| GLB(g/L) | 20.91 | 2.256 | 22.29 | | 4.237 | | 21.59 | 2.709 |
| pre-ALB(g/L) | 240.5 | 44.76 | 262.8 | | 110.7 | | 233.1 | 67.71 |
| TG (mmol/L) | 1.076 | 0.3279 | 2.143 | | 0.5751 | | 2.21 | 0.4188 |
| TC (mmol/L) | 3.882 | 0.6213 | 4.722 | | 0.8412 | | 5.036 | 0.9317 |

M/F, male/female; BMI, body mass index; PLT, platelets; INR, international normalized ratio; PT, prothrombin time; ALT, alanine aminotransferase; AST, aspartate aminotransferase; ALP, alkaline phosphatase; γGT, glutamyl transtitanase; TBil, total bilirubin; ALB, albumin; GLB, globulin; TG, triglyceride; TC, total cholesterol.

**Table S4** Primer sequences used for RT-quantitative PCR in this study

| Gene | Forward Primer（5’-3’） | Reverse Primer（5’-3’） |
| --- | --- | --- |
| *MCU* | GAGCCGCATATTGCAGTACG | CGAGAGGGTAGCCTCACAGAT |
| *Acc1* | GATGAACCATCTCCGTTGGC | GACCCAATTATGAATCGGGAGTG |
| *β-Actin* | GGCTGTATTCCCCTCCATCG | CCAGTTGGTAACAATGCCATGT |
| *Tnfα* | CCCTCACACTCAGATCATCTTCT | GCTACGACGTGGGCTACAG |
| *Il6* | TTAAAAACCTGGATCGGAACCAA | GCATTAGCTTCAGATTTACGGGT |
| *Il1β* | GCAACTGTTCCTGAACTCAACT | ATCTTTTGGGGTCCGTCAACT |
| *Mcp1* | TTAAAAACCTGGATCGGAACCAA | GCATTAGCTTCAGATTTACGGGT |
| *Srebp1* | TGACCCGGCTATTCCGTGA | CTGGGCTGAGCAATACAGTTC |
| *Fasn* | GGAGGTGGTGATAGCCGGTAT | TGGGTAATCCATAGAGCCCAG |
| *Pparγ* | TCGCTGATGCACTGCCTATG | GAGAGGTCCACAGAGCTGATT |
| *Col1α1* | GCTCCTCTTAGGGGCCACT | CCACGTCTCACCATTGGGG |
| *Col4α1* | CTGGCACAAAAGGGACGAG | ACGTGGCCGAGAATTTCACC |
| *Ctgf* | GGGCCTCTTCTGCGATTTC | ATCCAGGCAAGTGCATTGGTA |
| *Pai1* | TCAGCCCTTGCTTGCCTC | TTCAGCCCTTGCTTGCCTC |
| *αSma* | GTCCCAGACATCAGGGAGTAA | TCGGATACTTCAGCGTCAGGA |
| *Ho1* | AAGCCGAGAATGCTGAGTTCA | GCCGTGTAGATATGGTACAAGGA |
| *Sod1* | AACCAGTTGTGTTGTCAGGAC | CCACCATGTTTCTTAGAGTGAGG |
| *Catalase* | AGCGACCAGATGAAGCAGTG | TCCGCTCTCTGTCAAAGTGTG |
| *Pgc1α* | TATGGAGTGACATAGAGTGTGCT | CCACTTCAATCCACCCAGAAAG |
| *Capt1a* | CTCCGCCTGAGCCATGAAG | CACCAGTGATGATGCCATTCT |

**Supplementary Figures**

**
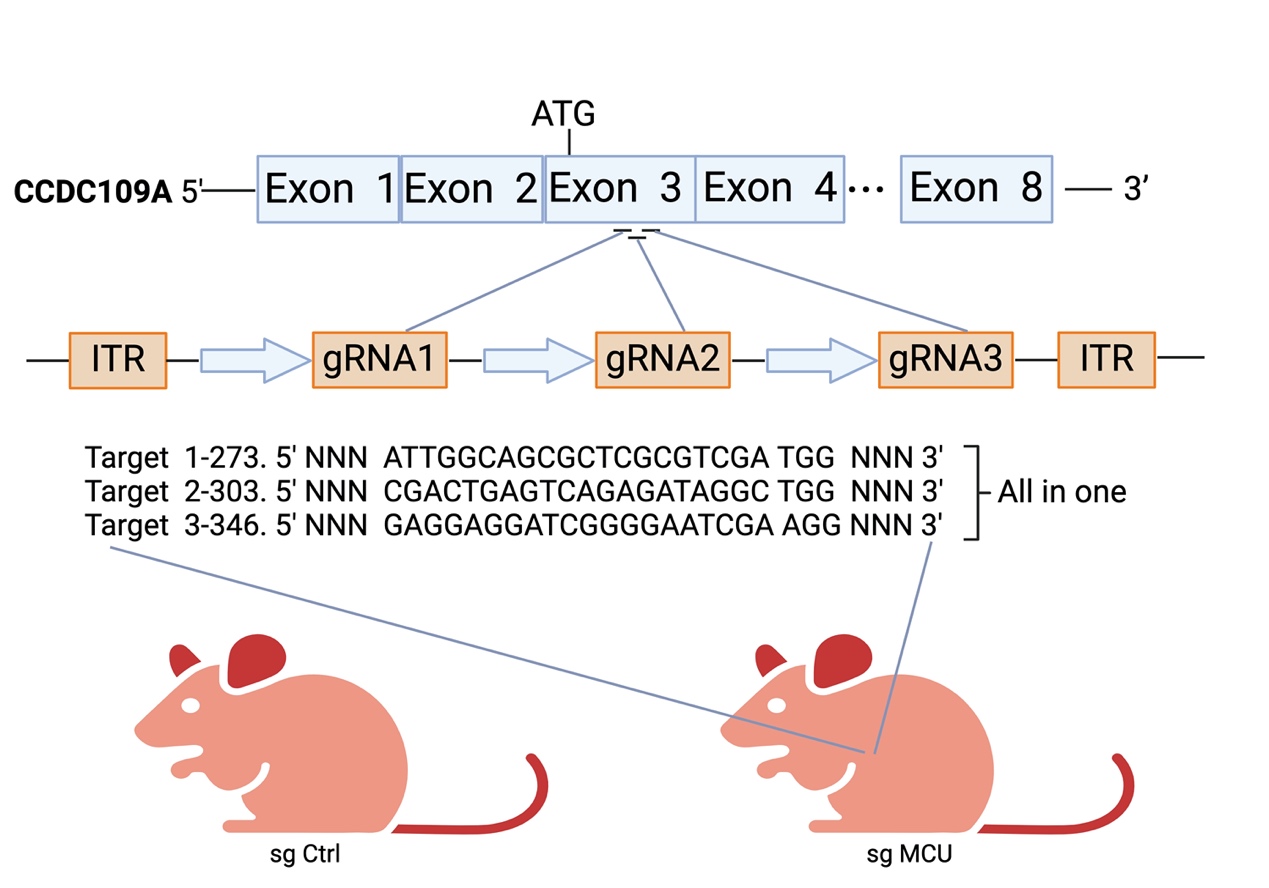
**

**Fig. S1** All-in-One AAV8 encoding small-guide RNA (sgRNA) targeting MCU.

**
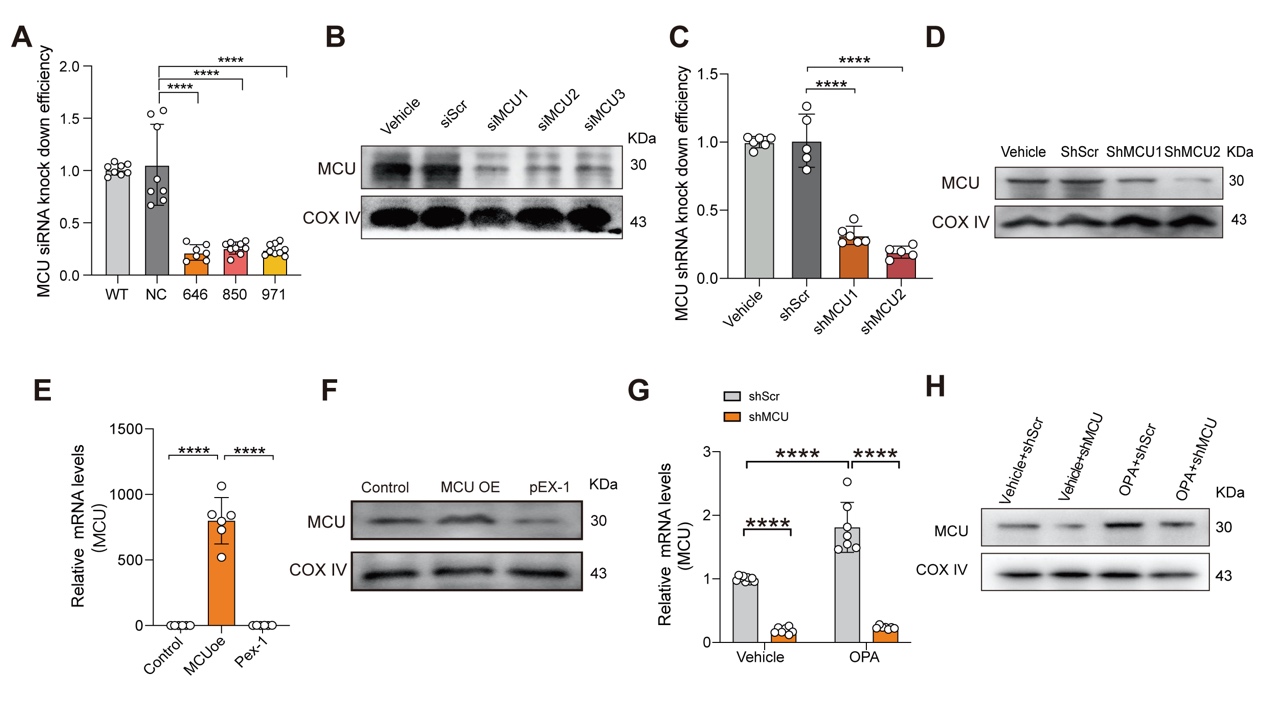
**

**Fig. S2** The verification and selection of MCU knockdown and overexpression.

**A, B** AML12 hepatocytes were transfected with non-targeted scrambled control siRNA (siScr) and 3 groups of siMCU (1, 2, and 3). After 24h of transfection, the knockdown efficiency of MCU siRNA was determined by qRT-PCR (**A**). The mRNA levels were normalized to β-actin mRNA levels and presented as fold stimulation versus control. After 48h of transfection, the knockdown efficiency of MCU siRNA was determined by western blot (**B**). COX IV served as a loading protein. With the highest knockdown efficiency, siMCU1 was finally chosen in this study. **C, D** AML12 hepatocytes were transfected with non-targeted scrambled control shRNA (shScr) and 2 groups of shMCU (1 and 2). The transfected cells were then passaged and screened by puromycin to obtain stable cell strains. The knockdown efficiency of MCU shRNA was determined by qRT-PCR (**C**). The mRNA levels were normalized to β-actin mRNA levels and presented as fold stimulation versus Control. Meanwhile, the knockdown efficiency of MCU shRNA was determined by western blot (**D**). COX IV served as a loading protein. With higher knockdown efficiency, shMCU2 was finally chosen in this study. **E** The overexpression efficiency of MCU determined by qRT-PCR. The mRNA levels were normalized to β-actin mRNA levels and presented as fold stimulation versus Control. F Western blots for MCU expression. **G** qRT-PCR analysis of MCU expression in AML 12 hepatocytes. Results were normalized to the β-actin mRNA. **H** Western blot for MCU protein expression in AML12 hepatocytes; results were normalized relative to expression of COX IV. Data are expressed as the mean ± SD. *P*-value are for two-way ANOVA with Sidak’s post-test (A, C, G), and two-tailed unpaired Student’s *t*-test (E). Statistical significance is denoted by **** *P* < 0.0001.


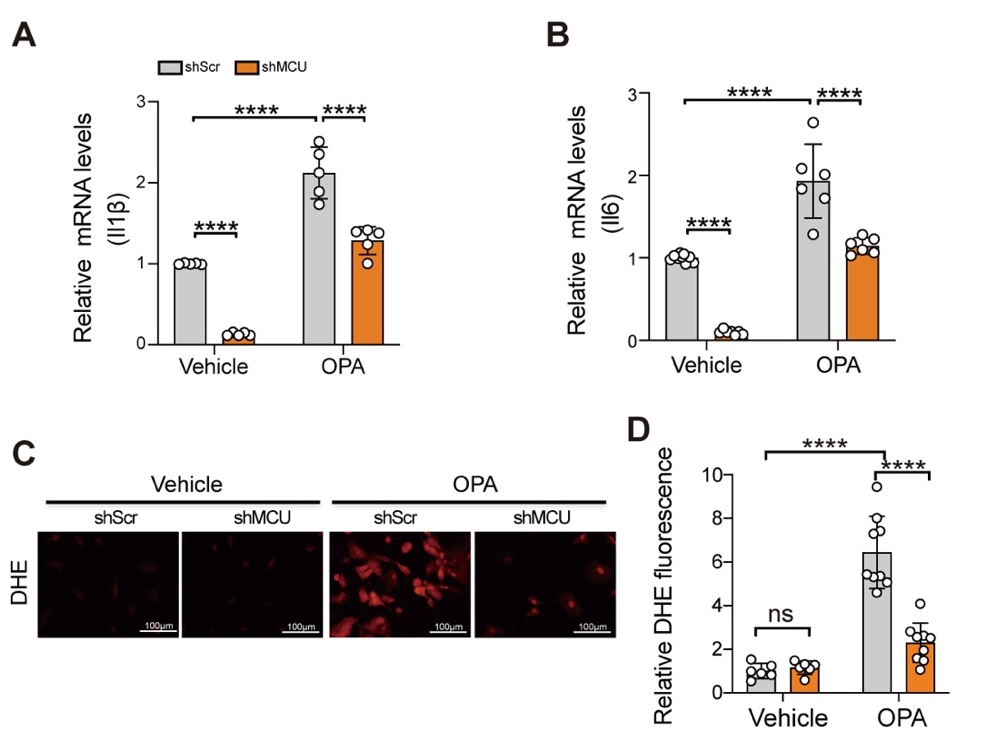


**Fig. S****3** MCU deficiency reduces inflammation in AML12 hepatocytes challenged with OPA.

**A, B** The levels of Il1β and Il6 mRNA expression in AML12 hepatocytes were measured by qRT-PCR. **C** Representative images of DHE staining in AML12 cells. Scale bars: 100 µm.**D** The intensity of red fluorescence of DHE oxidation was quantified (n = 6-9).The mRNA levels were normalized to β-actin mRNA levels and presented as fold stimulation versus Control. Data are mean ± SD. Two-way ANOVA with Tukey’s post-test (A), two-way ANOVA with Sidak’s test (B, D). Statistical significance is denoted by **** *P* < 0.0001.

**
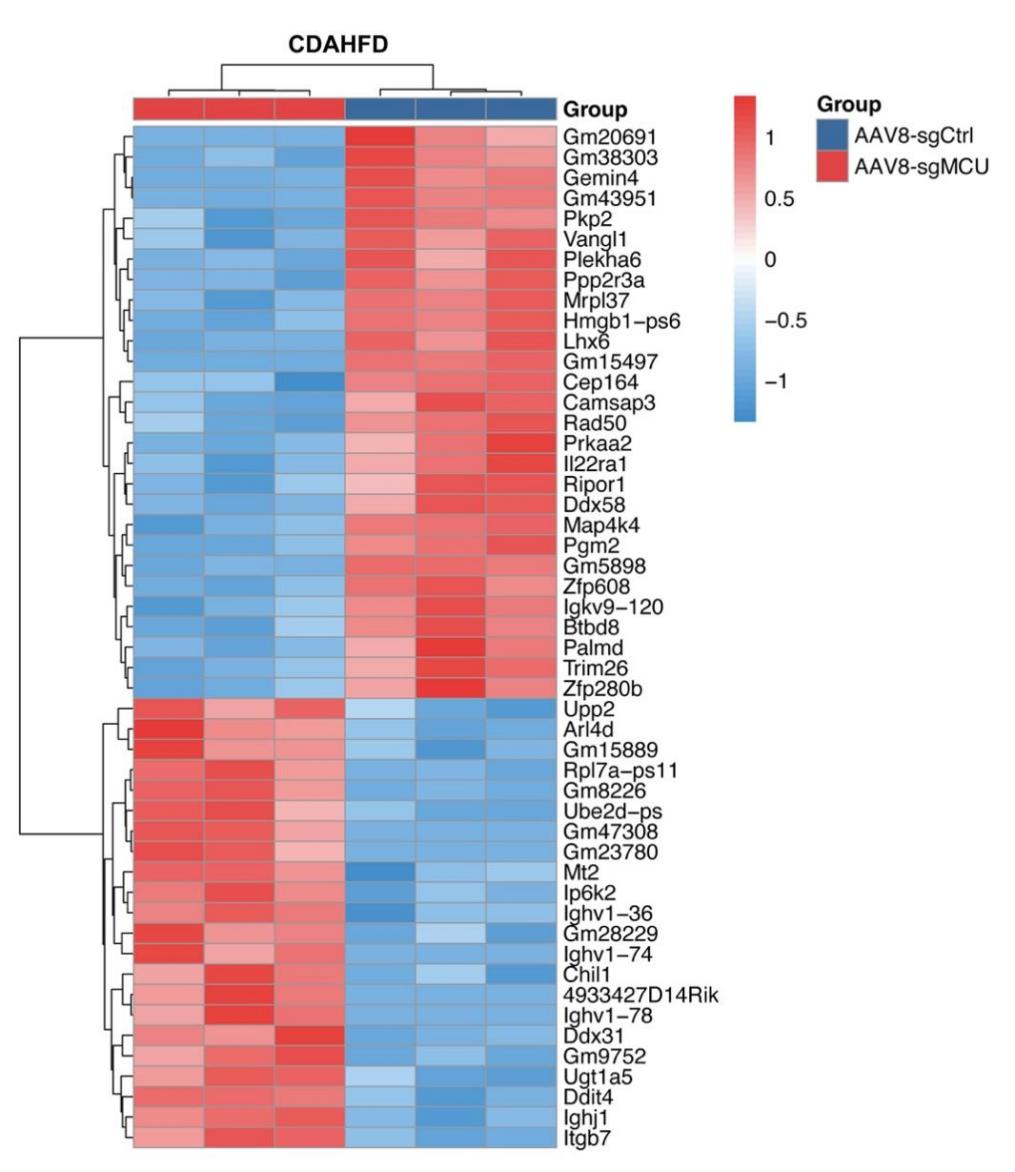
**

**Fig. S4** The top 50 genes that were up- or down-regulated analyzed from RNA-seq datesets. MCU knockout and wild-type (WT) mice were fed a Choline-deficient, L-amino acid-defined high-fat diet (CDAHFD) for 8 weeks. Color-coded as log2 (fold change) from -1 to 1 with *P* <0.05.

**
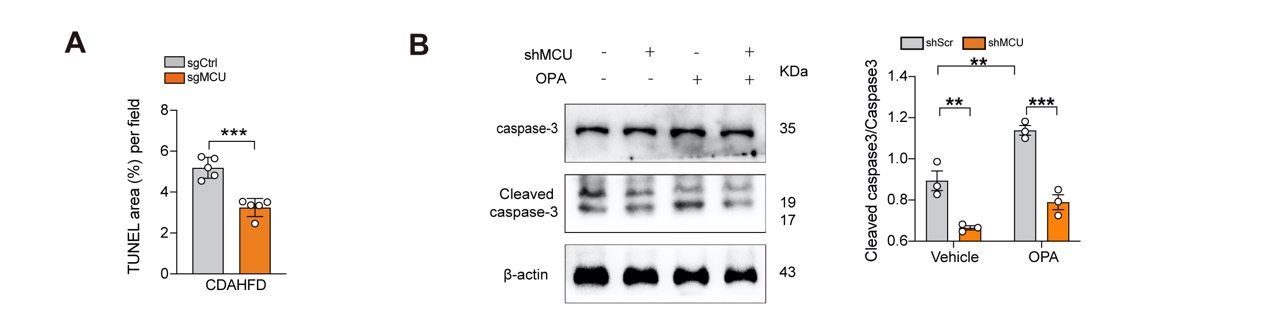
**

**Fig. S5** MCU drives hepatocyte death during MAFLD/MASH development both in mice and in vitro. **A** Quantification of TUNEL^+^ cells in liver sections from NASH mice (corresponding to Fig.7D; n=5). **B** Western blots for cellular extracts caspase-3, cleaved caspase-3, and analyses of blots; β-actin served as a loading protein, and densitometric analyses of blot for cleaved caspase-3/ caspase-3. Data are expressed as mean ± SD. *P*-values are for two-tailed unpaired Student’s *t*-test and two-way ANOVA with Tukey’s post-test. ***P* < 0.01, *** *P* < 0.001.


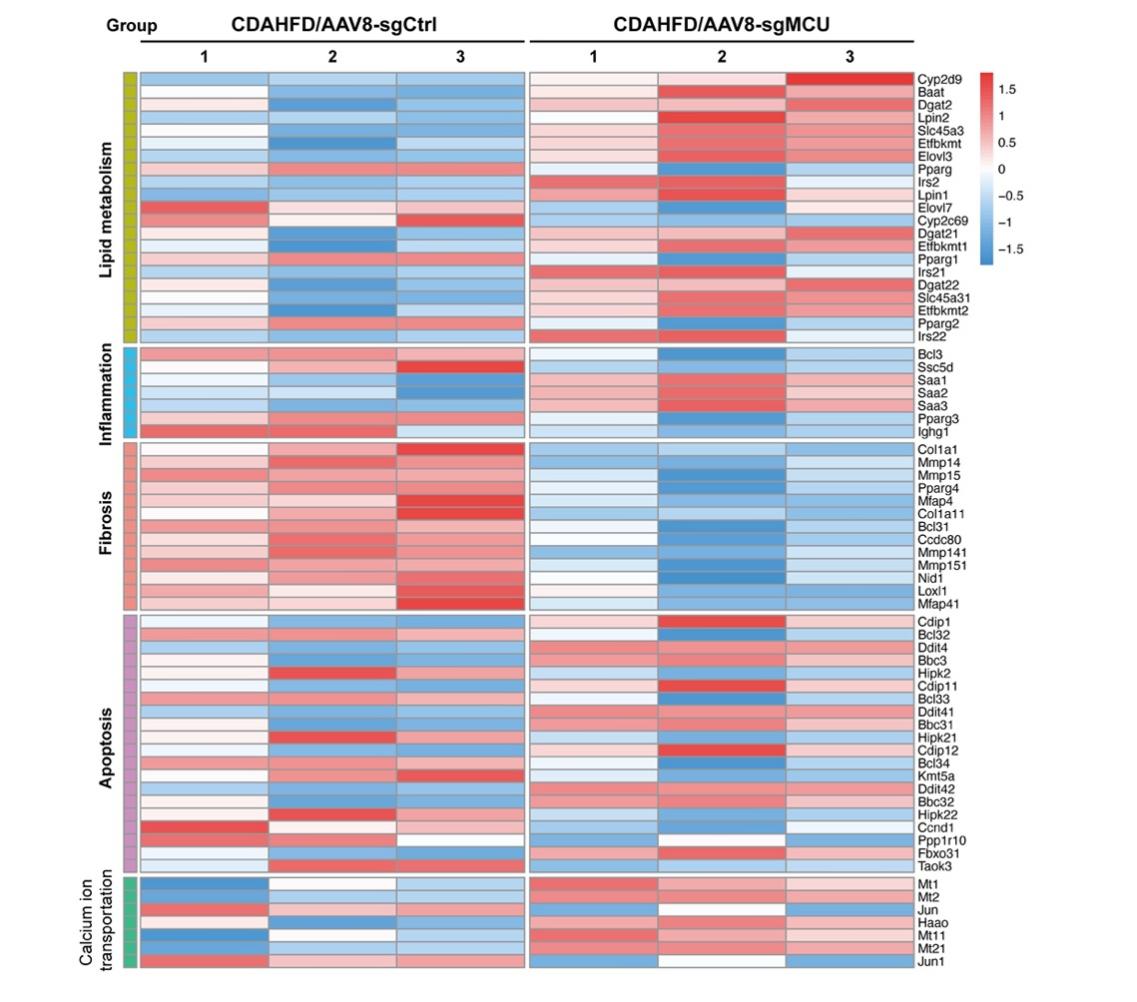


**Fig. S6** Heatmap showing the significantly altered genes related to lipid metabolism, inflammation, fibrosis, apoptosis, and calcium ion transportation.

Heatmap showing the significantly altered genes related to lipid metabolism, inflammation, fibrosis, apoptosis, and calcium ion transportation from RNA-seq data sets (n = 3 mice per group). Color-coded as log2 (fold change) from -1.5 to 1.5 with *P* <0.05. MCU knockout and wild-type (WT) mice were fed a Choline-deficient, L-amino acid-defined high-fat diet (CDAHFD) for 8 weeks.

**
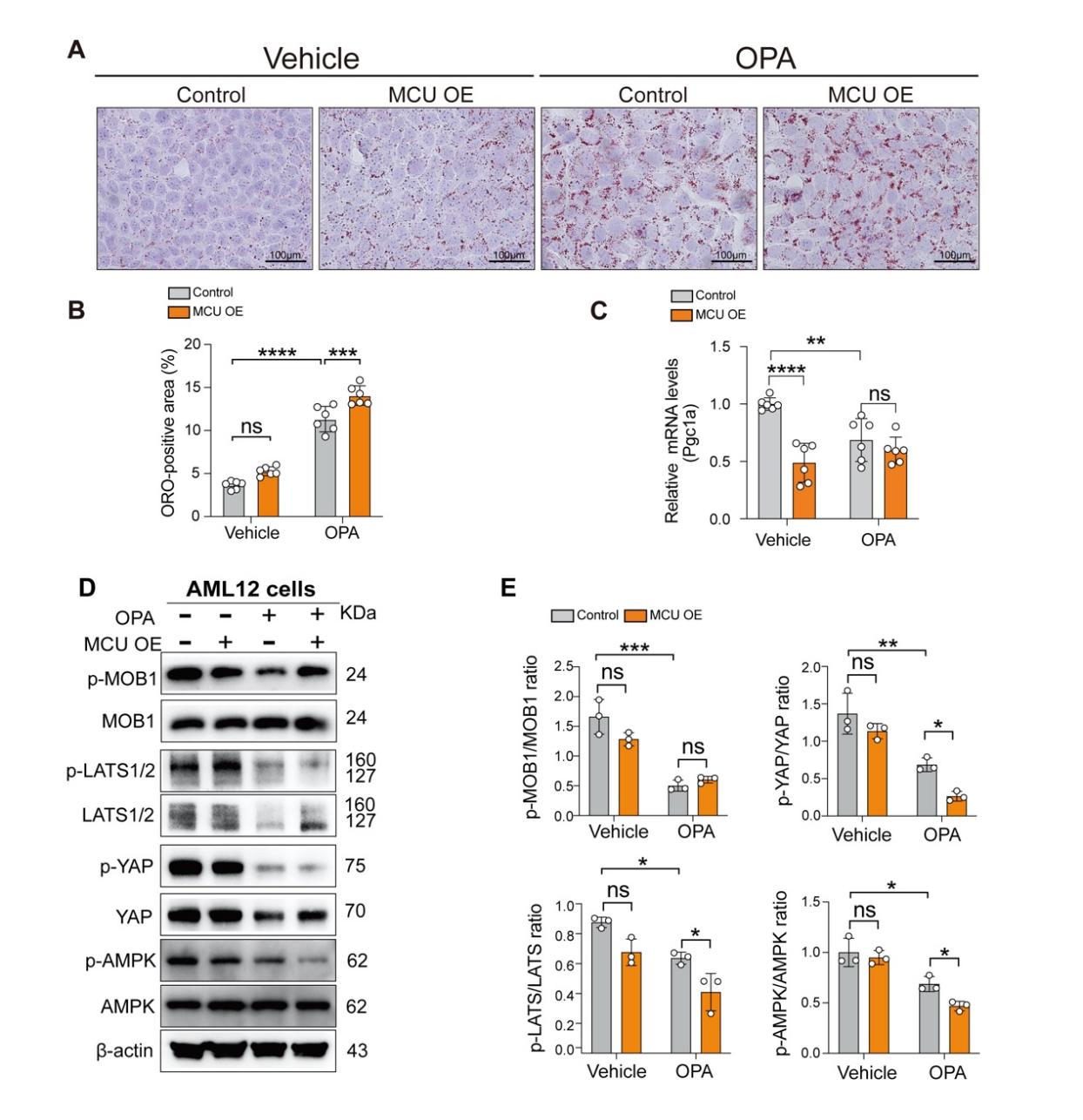
**

**Fig. S7** MCU overexpression in AML12 hepatocytes aggravated increased lipid deposition and activated the Hippo/YAP signaling in response to OPA overload.

For overexpression of MCU, AML12 hepatocytes were transfected with empty pEX-1 vector or pEX-1-MCU plasmid (MCU OE). After 48 h of transfection, the cells were treated with OPA or vehicle for 24 h. **A** Representative images of ORO staining in AML12 hepatocytes. Scale bar: 100 µm. **B** The quantification of ORO^+^ area in AML12 hepatocytes by software ImageJ. **C** Levels of Pgc1α mRNA expression determined by qRT-PCR. **D** Western blots for p-MOB1, MOB1, p-LATS1/2, LATS1/2, p-AMPK/AMPK, p-YAP and YAP, and β-actin as loading control. **E** Densitometric analyses of blots for p-MOB1/MOB1, p-YAP/YAP, p-LATS/LATS, and p-AMPK/AMPK. All data are presented as the mean ± SD. *P*-values are for two-way ANOVA with Tukey’s test. **P* < 0.05, ***P* < 0.01, and ****P* < 0.001, ****P* < 0.001; ns indicates not significant.

**
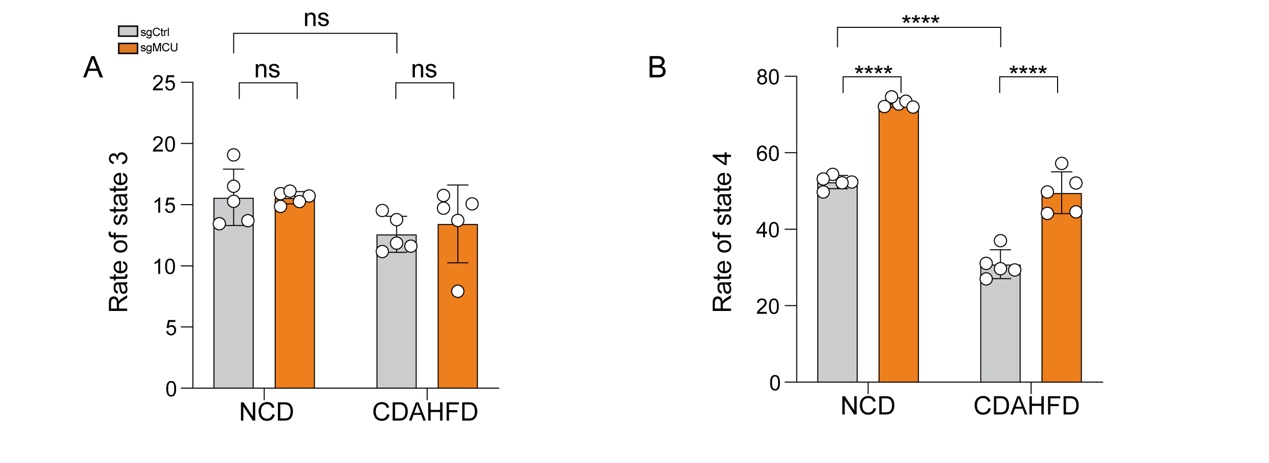
**

**Fig. S8** The rate of state 3 and state 4 in the liver mitochondria isolated from indicated mice.

**A** The rate of state 3 indicates MCU expression has no significant effect on the damage in the electron transport chain. **B** The rate of state4 indicates MCU expression affects the leakage in the mitochondrial inner membrane. *P*-values are for two-way ANOVA with Tukey’s test. *****P* < 0.001; ns indicates not significant.
