# Supplementary material for: Knockdown of hepatic mitochondrial calcium uniporter mitigates MASH and fibrosis in mice
Source: Cell Biosci. 2024 Nov 10;14:135. doi: 10.1186/s13578-024-01315-4 (PMC11550531; doi:10.1186/s13578-024-01315-4)
Supplement: Supplementary file 2 — Supplementary Material 2 [file 13578_2024_1315_MOESM2_ESM.docx]

FIGURE2 B

MCU

COX IV

FIGURE 2 E

MCU

COX IV

FIGURE 4 B

MCU

COX4

FIGURE 5 F

α-SMA

β-actin

FIGURE 7 I

Cleaved-caspase3

Total Caspase3

β-actin

FIGURE 9 C

p-MOB1

MOB1

p-LATS1/2

LATS1/2

p-YAP

YAP

p-AMKP

Ampk

β-actin

FIGURE 10 A

p-MOB1

MOB1

p-LATS

LATS

p-YAP

YAP

p-AMPK

AMPK

β-actin

FIGURE 9 D

p-MOB1

MOB1

p-LATS1/2

LATS1/2

p-YAP

YAP

β-actin

FIGURE S2 B

MCU

COX4

FIGURE S2 D

MCU

COX IV

FIGURE S2 F

MCU

COX4

FIGURE S2 H

MCU

COX IV

FIGURE S5 B

Caspase3

Cleavedcaspase3

Actin

FIGURE S7D

p-MOB1

MOB1

p-LATS1/2

LATS1/2

p-YAP

YAP

p-AMPK

AMPK

β-actin
